# Effects of repetitive transcranial magnetic stimulation on gait disorders and cognitive dysfunction in Parkinson's disease: A systematic review with meta‐analysis

**DOI:** 10.1002/brb3.2697

**Published:** 2022-07-21

**Authors:** Shan Deng, Zhimei Dong, Liya Pan, Ying Liu, Ziming Ye, Lu Qin, Qianqian Liu, Chao Qin

**Affiliations:** ^1^ Department of Neurology The First Affiliated Hospital of Guangxi Medical University Nanning China; ^2^ Department of Neurology The Fourth Affiliated Hospital of Guangxi Medical University Liuzhou China

**Keywords:** cognitive dysfunction, freezing of gait, meta‐analysis, Parkinson's disease, transcranial magnetic stimulation

## Abstract

**Background:**

Repetitive transcranial magnetic stimulation (rTMS) is acknowledged to be crucial to manage freezing of gait (FOG) and cognitive impairment for patients with Parkinson's disease (PD), but its effectiveness is unclear.

**Objective:**

To determine the effects of rTMS on FOG and cognitive function in people with PD and to investigate potential factors that modulate the rTMS effects.

**Methods:**

Databases searched included PubMed, Web of Science, EMBASE, and the Cochrane Library from inception to December 31, 2021. Eligible studies include a controlled randomized clinical trial of rTMS intervention for FOG and cognitive dysfunction in PD patients. The weighted mean difference (WMD) with 95% confidence intervals (CI) were calculated with fixed‐effects models. The outcome of the study included gait and cognitive assessments.

**Results:**

Sixteen studies with a total of 419 patients were included. Fixed‐effects analysis revealed that rTMS was effective in improving freezing of gait questionnaire scores (short‐term effect: WMD = −0.925, 95% CI: −1.642 to −0.209, *p* = .011; long‐term effect: WMD = −2.120, 95% CI: −2.751 to −1.489, *p* = .000), 10‐m walking time (short‐term effect: WMD = −0.456, 95% CI: −0.793 to −0.119, *p* = .008; long‐term effect: WMD = −0.526, 95% CI: −0.885 to −0.167, *p* = .004), Timed Up‐and‐Go scores (short‐term effect: WMD = −1.064, 95% CI: −1.555 to −0.572, *p* = .000; long‐term effect: WMD = −1.097, 95% CI: −1.422 to −0.772, *p* = .000), Montreal cognitive assessment (WMD = 3.714, 95% CI: 2.567 to 4.861, *p* = .000), and frontal assessment battery (WMD = −0.584, 95% CI: −0.934 to −0.234, *p* = .001).

**Conclusions:**

RTMS showed a beneficial effect on FOG and cognitive dysfunction in parkinsonism. However, the optimal rTMS protocol has not been determined and further high‐quality studies are needed.

## Graphical Abstract

Repetitive transcranial magnetic stimulation was effective in improving the FOG‐Q scores, 10 m walking time, TUG scores, MOCA, and FAB.Repetitive transcranial magnetic stimulation showed a beneficial effect on FOG and cognitive dysfunction in parkinsonism.

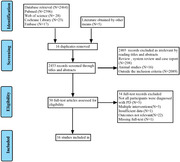

## 1 INTRODUCTION

Parkinson's disease (PD) is a common progressive neurodegenerative disorder. In addition to motor symptoms such as rigidity, bradykinesia, postural instability, and gait disturbances, people with PD often present a range of nonmotor symptoms such as cognitive dysfunction, depression, and autonomic dysfunction (Khoo et al., 2013). It is well established that the degeneration of dopaminergic neurons will lead to symptoms that are difficult to cure with conventional treatment. Among them, freezing of gait (FOG) and cognitive dysfunction are common, cause disability, and reduce the quality of life in advanced PD.

FOG is defined as “brief, episodic absence or marked reduction of forward progression of the feet despite the intention to walk” (Bloem et al., 2004; Giladi & Nieuwboer, 2008). Some studies (Giladi et al., 1992, 2001; Lamberti et al., 1997) have shown that freezing is present in about 7% of people with PD in the first 2 years of the disease, 28% at 5 years, 39% at 10 years, and 58% after 10 years. The profile of cognitive dysfunction in PD patients is heterogeneous. The degree of cognitive deterioration in PD is variable and ranges from mild cognitive impairment to dementia. There is also variation in the number and type of affected cognitive domains which can involve either a single domain like executive or visuospatial function or multiple ones (Koros et al., 2022). Cognitive disorder is general even in the early stages of PD (Foltynie et al., 2004), and nearly 80% of patients with mild cognitive impairment eventually develop dementia later in the disease (Caviness et al., 2007). A study indicated that FOG is related to cognitive impairment in patients with PD (Yao et al., 2017). FOG and cognitive impairment are linked through mutual causality, and both conditions worsen as the PD disease progresses (Vandenbossche et al., 2012). Drug therapy is the first choice for PD combined with either FOG or cognitive impairment, but the effect is limited. The effects of medication diminish over time, and side effects become apparent. Deep brain stimulation is not suitable for all patients with PD and only improves DOPA‐responsive FOG. Therefore, there is an urgent clinical need to explore and find new treatments for PD combined with FOG or cognitive impairment.

Transcranial magnetic stimulation (TMS) is a noninvasive neuromodulation technique based on Faraday's principle of electromagnetic induction (Lefaucheur et al., 2014). TMS can induce electrical currents and alter cortical activity in the human brain by delivering strong magnetic pulses to the brain regions (Lefaucheur et al., 2014; Ni & Chen, 2015). Repetitive TMS (rTMS) refers to the application of trains of regularly repeating TMS pulses that modulate neural excitability and cortical function (Valero‐Cabré et al., 2017). The parameters of rTMS include frequency, stimulation location, intensity, number of pulses, interval time, and duration of treatment. Previous studies have shown that low frequencies (≤1 Hz) of rTMS induce suppression of cortical excitability (Gangitano et al., 2002), whereas higher frequencies (≥5 Hz) have the opposite effect (Berardelli et al., 1998). Since the clinical application of TMS was first reported in 1985 (Barker et al., 1985), it has become widely used to treat various neurological and psychiatric disorders, including PD and Alzheimer's disease (Lefaucheur et al., 2020).

Although there is growing evidence that rTMS has a positive effect on FOG and cognitive function in patients with PD (Chang et al., 2017; Kim et al., 2015; Mi et al., 2019; Trung et al., 2019; Zhuang et al., 2020), some studies have not found such an effect (Benninger et al., 2011, 2012; Cohen et al., 2018). However, there is significant heterogeneity in the study design, stimulus settings, and participants between the studies. Therefore, the protocol and parameter design of rTMS for PD needs to be further investigated. Several meta‐analyses (Goodwill et al., 2017; P. K. He et al., 2020; Kim et al., 2019; Lawrence et al., 2017; Xie et al., 2020) have recently evaluated the therapeutic effects of rTMS on FOG, cognition in patients with PD; however, their conclusions were inconsistent. Recently, four new randomized controlled trials (Cheng et al., 2022; W. He et al. 2021; Mi et al., 2020; Zhuang et al., 2020) have been published, providing an opportunity to update the meta‐analysis in this area. This study builds on previous meta‐analyses and aims to assess the therapeutic effects of rTMS on FOG and cognition in patients with PD and provide updated evidence on the role of rTMS therapy in patients with PD.

## 2 METHOD

This protocol is registered on PROSPERO (registration number is CRD42022303267). This study was reported in accordance with the Preferred Reporting Items for Systematic Reviews and Meta‐Analyses (PRISMA) statement (Moher et al., 2009).

### 2.1 Search strategy and inclusion/exclusion criteria

**FIGURE 1:**
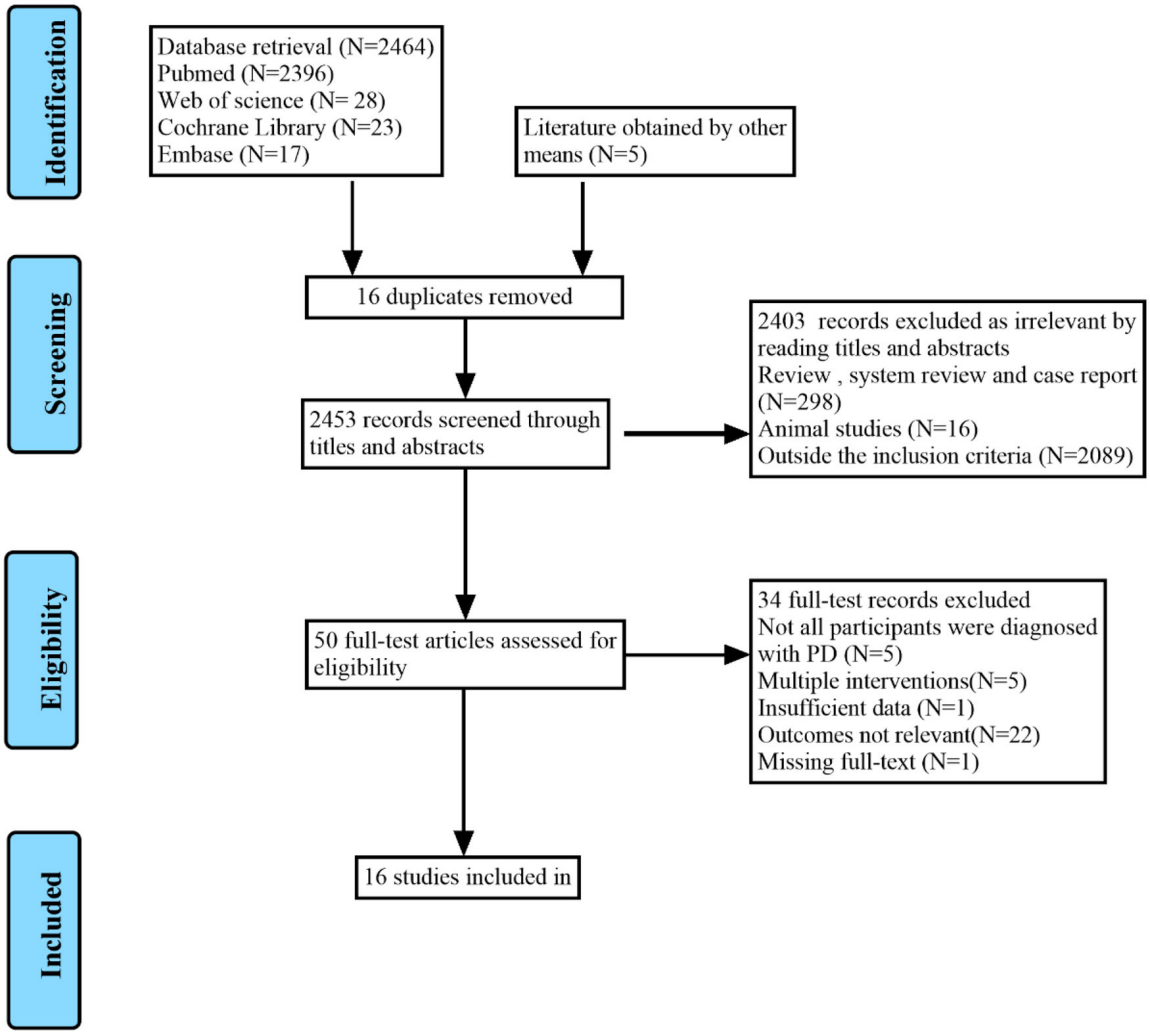
PRISMA flow diagram

We conducted a systematic search of the literature in the PubMed, EMBASE, Web of Science, and Cochrane Library databases from the earliest available record until December 31, 2021, without restriction to regions, publication types, or languages. In addition, a list of references of relevant studies was reviewed to identify potential studies. The search process is described in Figure 1. The following search terms were used: (“Parkinson Disease” OR “Parkinson's Disease” OR “Parkinsonism” OR “Paralysis Agitans” OR “PD”) AND (“Gait Disorders, Neurologic” OR “Gait Disorder” OR “Locomotion Disorders” OR “Neurologic Ambulation Disorders” OR “Gait Dysfunction, Neurologic” OR “motor function” OR “freezing of gait” OR “gait impairments” OR “FOG”) AND (“Cognition” OR “Cognitive Function” OR “Cognitive Dysfunction” OR “Cognition Disorders” OR “Cognitive Impairment” OR “Neurocognitive Disorder” OR “Cognitive Decline” OR “Dementia” OR “MCI” OR “PDD”) AND (“transcranial magnetic stimulation” OR “repetitive transcranial magnetic stimulation” OR “TMS” OR “rTMS”) AND (“randomized controlled trial” OR “randomized” OR “placebo” OR “RCT”). The complete search strategies of the three electronic databases are provided in Table S1.

Trials that met all the following criteria (PICOS) were included: (1) “P”: The patients in the study were diagnosed with idiopathic PD according to the British Parkinson's Society Brain Bank or the Movement Disorders Society Clinical Diagnostic Criteria for Parkinson's Disease (Litvan et al., 2012); (2) “I”: Intervention using rTMS; the study provided a detailed description of the intervention, such as stimulation site, intensity, frequency, and time; (3) “C”: rTMS sham stimulation or dual‐mode noninvasive brain stimulation (NIBS) as control; (4) “O”: The outcome of the study included gait, cognitive assessments, such as freezing of gait questionnaire (FOG‐Q), 10‐m walking time, timed up‐and‐go test (TUG), Montreal cognitive assessment (MOCA), and frontal assessment battery (FAB); (5) “S”: The study used randomized parallel or crossover design with a controlled group; (6) Data to calculate effect sizes (pre‐post means, standard deviations, and sample sizes) were provided.

The exclusion criteria included: (1) case reports, animal experiments, review, and system review; (2) no control group; (3) no data available to determine effect sizes; (4) studies using rTMS combined with another therapy; (5) duplicated studies published by the same research group were excluded. Studies were excluded if they were a conference abstract or lacked the data required for estimating the effect size. If the included studies lacked key information, we contacted the corresponding authors for further information about their trials.

### 2.2 Data collection and extraction

Two authors (Shan Deng and Zhimei Dong) independently searched the titles and abstracts of the articles to assess eligibility for inclusion. The full texts of the eligible articles were reviewed and subsequently evaluated. The two authors independently extracted the following data from the included studies: (1) characteristics of the studies: author, year of publication, sample size, and study design; (2) participant demographics (country, age, gender, on or off state, duration of disease, and severity of disease), stimulation protocols (site, frequency, number of pulses, intensity, and duration), and the test used to quantify FOG and cognitive function outcome measures. Tables 1 and 2 show the extracted data in detail. Disagreements were resolved by discussion between the two authors. If further clarification was required, Professor Chao Qin was consulted. For each included study, quantitative data were extracted from the outcome text, tables, and graphs for the pre‐ and postintervention, stimulus, and control (sham) conditions. If the studies reported more than one “post” assessment, the time points after the first and last intervention were extracted and used for analysis. In two‐phase crossover studies, data from the end of the first phase (treatment vs. placebo control) prior to the crossover studies were used.

**TABLE 1:** Study participant characteristics of the included trials

			*N*		Mean age, years, SD		Sex, M (F)		DD, MEan years, SD		H‐Y Stage	
Trial (Year)	Country	Study design	Experimental group	Control group	Experimental group	Control group	Experimental group	Control group	Experimental group	Control group	Experimental group	Control group
Benninger et al. (2011).	USA	RCT	13	13	62.1 ± 6.9	65.6 ± 9.0	7 (6)	11 (2)	10.8 ± 7.1	6.5 ± 3.4	2.6 ± 0.2	2.5 ± 0.1
Chang et al. (2017)	Korea	RCT	16	16	63.8 ± 8.3	63.6 ± 7.5	11 (5)	9 (7)	9.1 ± 5.3	9.8 ± 4.7	N/A	N/A
Cohen et al. (2018)	Israel	RCT	21	21	64.4 ± 6.8	66.8 ± 8.1	17 (4)	15 (6)	4.7 ± 3.4	5.6 ± 3.7	2.0 (2.0−2.5)	2.0 (2.0−2.5)
Benninger et al. (2012)	USA	RCT	13	13	64.5 ± 9.1	63.7 ± 8.3	11 (2)	9 (4)	8.6 ± 4.1	9.3 ± 6.8	2.4 ± 0.2	2.5 ± 0.3
Dagan et al. (2017)	Israel	RCD	7	7	74.57 ± 7.09	74.57 ± 7.09	7 (0)	7 (0)	10.29 ± 3.82	10.29 ± 3.82	2.86 ± 0.63	2.86 ± 0.63
Kim et al. (2015)	Korea	RCD	17	17	64.5 ± 8.4	64.5 ± 8.4	12 (5)	12 (5)	7.8 ± 4.9	7.8 ± 4.9	3.0 ± 0.5	3.0 ± 0.5
Maruo et al. (2013)	Japan	RCD	10	11	63.0 ± 11.3	63.0 ± 11.3	11 (10)	11 (10)	12.0 ± 6.3	12.0 ± 6.3	3.1 ± 0.5	3.1 ± 0.5
Mi et al. (2019)	China	RCT	20	10	62.65 ± 10.56	65.60 ± 8.68	9 (11)	5 (5)	9.15 ± 5.82	7.40 ± 4.83	2.60 ± 0.85	2.35 ± 0.91
Zhuang et al. (2020)	China	RCT	19	14	60.58 ± 9.21	61.57 ± 13.25	11 (8)	7 (7)	5.86 ± 4.36	5.71 ± 3.77	2 (1.5,2.5)	2.25 (1.75, 3.0)
Ma et al. (2019)	China	RCT	18	10	59.94 ± 9.16	66.00 ± 8.55	8 (10)	5 (5)	8.94 ± 5.48	7.50 ± 4.72	2.42 ± 0.60	2.40 ± 0.94
Mi et al. (2020)	China	RCT	20	10	62.65 ± 10.56	65.60 ± 8.68	9 (11)	5 (5)	9.15 ± 5.82	7.40 ± 4.83	2.60 ± 0.85	2.35 ± 0.91
Srovnalova et al. (2011)	Czech	RCD	10	10	66 ± 6.0	66 ± 6.0	6 (4)	6 (4)	5.4 ± 2.45	5.4 ± 2.45	N/A	N/A
Pal et al. (2010)	Hungary	RCT	12	10	68.5 ± ?	67.5 ± ?	6 (6)	5 (5)	6.0 ± ?	6.5 ± ?	N/A	N/A
Khedr et al. (2020)	Egypt	RCT	18	15	65.56 ± 8.73	59.33 ± 10.27	14 (4)	10 (5)	5.89 ± 5.37	5.50 ± 3.85	N/A	N/A
W. He et al. (2021)	Hong Kong, Taiwan	RCT	20	15	70.0 ± 6.3	74.8 ± 6.9	13 (7)	10 (5)	2.7 ± 1.5	2.5 ± 1.1	2.7 ± 1.1	2.5 ± 1.0
Cheng et al. (2021)	Taiwan	RCT	11	16	71.6 ± 5.1	73.9 ± 6.9	6 (5)	11 (5)	N/A	N/A	3.0 ± 1.2	2.5 ± 1.0

Abbreviations: DD, disease duration; F, female; H‐Y Stage, Hoehn and Yahr Stage; M, male; N/A, not applicable; RCD, randomized cross‐over design; RCT, randomized controlled trial; SD, standard deviation.

**TABLE 2:** Characteristics of included studies: RTMS variables

Trial (year)	rTMS site	rTMS frequency, Hz	Intensity	No. of pulses per session	Total sessions of rTMS	Control measures	Post‐rTMS evaluation	On/off (evaluation)	Outcome measures
Benninger et al. (2011).	Bilateral M1, DLPFC	ITBS (50 Hz→5 Hz)	80%AMT	1200	8	Sham iTBS	1 day, 30 days	On	10‐m walking time, FAB
Chang et al. (2017)	M1‐LL	10 Hz	90% RMT	1000	5	Dual‐mode NIBS	5 days, 12 days	On	FOG‐Q, TUG, MoCA
Cohen et al. (2018)	Bilateral M1, PFC	1 Hz and 10 Hz	110%MT and 100%MT	1700	24	Sham	90 days	On	TUG
Benninger et al. (2012)	Bilateral M1	50 Hz	80%AMT	720	8	Sham	1 day, 30 days	On	10‐m walking time, FOG‐Q, FAB
Dagan et al. (2017)	Bilateral PFC	10 Hz	100% RMT	2100	16	Sham	56 days	On	FOG‐Q
Kim et al. (2015)	M1‐LL	10 Hz	90% RMT	1000	5	Sham	1 day, 12 days	On	FOG‐Q, TUG
Maruo et al. (2013)	Bilateral M1 foot area	10 Hz	100% RMT	1000	3	Sham	4 days, 14 days	On	10‐m walking time
Mi et al. (2019)	SMA	10 Hz	90% RMT	1000	10	Sham	14 days, 42 days	On	FOG‐Q
Zhuang et al. (2020)	The right DLPFC	1 Hz	110% RMT	1200	10	Sham	1 day, 30 days	On	MoCA
Ma et al. (2019)	SMA	10 Hz	90% RMT	1000	10	Sham	10 days, 28 days	On	FOG‐Q
Mi et al. (2020)	SMA	10 Hz	90% RMT	1000	10	Sham	10 days, 28 days	On	TUG, FOG‐Q
Srovnalova et al. (2011)	Bilateral IFG	25 Hz	80% RMT	600	1	Sham	1 day	On	FAB
Pal et al. (2010)	The left DLPFC	5 Hz	90% RMT	600	10	Sham	1 day, 40 days	On	TUG
Khedr et al. (2020)	Bilateral M1	20 Hz	90% RMT	2000	10	Sham	1 day	N/A	MoCA
W. He et al. (2021)	The left DLPFC	ITBS	100% RMT	N/A	10	Sham	10 days	On	MoCA
Cheng et al. (2021)	The left DLPFC	ITBS (50 Hz→5 Hz)	90% RMT	600	10	Sham	1 day	On	MoCA

Abbreviations: AMT, active motor threshold; DLPFC, dorsolateral prefrontal cortex; EDB, extensor digitorum brevis motor representation; FAB, Frontal Assessment Battery; FOG‐Q, freezing of gait questionnaire; IFG, inferior frontal gyri; iTBS, intermittent theta‐burst stimulation; M1, the primary motor; M1‐LL, primary motor cortex of the lower leg; MEP, motor evoked potential; MoCA, Montreal Cognitive Assessment; MT, motor threshold; N/A, not applicable; NIBS, non‐invasive brain stimulation; PFC, the prefrontal cortex; RMT, resting motor threshold; SMA, the supplementary motor area; TUG, timed up‐and‐go.

### 2.3 Quality assessment

Two authors (Shan Deng and Zhimei Dong) independently assessed the methodological quality of the included trials using the risk of bias tool in the Cochrane Handbook for the Systematic Evaluation of Interventions, version 5.1.0. The tool reviews six potential sources of bias, including sequence allocation, allocation concealment, participant and personnel blinding, incomplete outcome data, selective outcome reporting, and other sources of bias. The risk of bias and quality of evidence were assessed as low, unclear (insufficient detail or not reported), or high, according to the Cochrane Handbook.

### 2.4 Statistical analysis

Stata version 14.0 (Stata Corp LP, USA) and RevMan5.3 software (The Nordic Cochrane Centre, The Cochrane Collaboration, Copenhagen, Denmark) were used to conduct the meta‐analysis. The weighted mean difference (WMD) with a 95% confidence interval (CI) was used to show the combined results. The Cochran's *Q* statistic and *I* ^2^ test were used to test for heterogeneity. If the value of *I* ^2^ was less than 50%, the fixed effects model was used for the analysis. Otherwise, a random model was used. Where necessary, a sensitivity analysis was carried out to check the robustness of the results. Funnel plots were assessed for publication bias using Egger's regression test (where non‐significant asymmetry indicated no bias). Outcome variables were compared using a value of *p* < .05 as statistically significant.

## 3 RESULTS

### 3.1 Results of the search

A total of 2469 studies were screened using the titles and abstracts, and another five references were obtained through other methods. The literature search identified 2453 references after the removal of duplicates. Of these, 2403 were excluded because they were not clinical trials or were outside the inclusion criteria. After full‐text screening, 34 studies were excluded because not all participants were diagnosed with PD, they involved multiple interventions, had insufficient data, had no standard gait or cognitive outcomes, or the full‐text was unavailable. Finally, 16 randomized controlled trials (Benninger et al., 2011, 2012; Chang et al., 2017; Cheng et al., 2022; Cohen et al., 2018; Dagan et al., 2017; W. He et al., 2021; Khedr et al., 2020; Kim et al., 2015; Ma et al., 2019; Maruo et al., 2013; Mi et al., 2019, 2020; Pal et al., 2010; Srovnalova et al., 2011; Zhuang et al., 2020) were included in the meta‐analysis. The reasons for noninclusion and exclusion at the full‐text screening stage are summarized in the PRISMA flow chart shown in Figure 1.

### 3.2 Characteristics of the included studies

The general characteristics of the included studies are shown in Tables 1 and 2. Studies were conducted in several countries. One was a multicenter study from Hong Kong and Taiwan (W. He et al., 2021).

Of the remaining studies, five were from China (Cheng et al., 2022; Ma et al., 2019; Mi et al., 2019, 2020; Zhuang et al., 2020), two were from the United States (Benninger et al., 2011, 2012), Korea (Chang et al., 2017; Kim et al., 2015) and Israel (Cohen et al., 2018; Dagan et al., 2017), and one was from Japan (Maruo et al., 2013), Hungary (Pal et al., 2010), Egypt (Khedr et al., 2020), and the Czech Republic (Srovnalova et al., 2011). The sample size ranged from seven to 42 (total number of participants was 419). The mean age of the study participants ranged from 59.94 ± 9.16 years to 74.57 ± 7.09 years, and 62.052% of the subjects were male. Among the 16 studies, 12 used a parallel‐group design, while the rest adopted a crossover design. The average disease duration for all 16 studies was between 2.5 ± 1.1 years and 12.0 ± 6.3 years, and the Hoehn and Yahr scale ranged from 2 to 3.1 ± 0.5.

Eleven studies administered high‐frequency TMS (> 1 Hz; range 5–50 Hz) (Benninger et al., 2012; Chang et al., 2017; Dagan et al., 2017; Khedr et al., 2020; Kim et al., 2015; Ma et al., 2019; Maruo et al., 2013; Mi et al., 2019, 2020; Pal et al., 2010; Srovnalova et al., 2011), one low (1 Hz) (Zhuang et al., 2020), and one study used both (Cohen et al., 2018). In addition, three studies used an intermittent theta‐burst stimulation (ITBS: 50 Hz → 5 Hz) protocol (Benninger et al., 2011; Cheng et al., 2022; W. He et al., 2021). The most common site of rTMS stimulation was the primary motor cortex (M1) (Benninger et al., 2011, 2012; Chang et al., 2017; Cohen et al., 2018; Khedr et al., 2020; Kim et al., 2015; Maruo et al., 2013) and the prefrontal cortex (PFC) (Benninger et al., 2011; Cheng et al., 2022; Cohen et al., 2018; Dagan et al., 2017; W. He et al., 2021; Pal et al., 2010; Zhuang et al., 2020). Three studies stimulated the supplementary motor area (SMA) (Ma et al., 2019; Mi et al., 2019, 2020) and one study stimulated the inferior frontal gyri (IFG) (Srovnalova et al., 2011). Of these, seven studies (Benninger et al., 2011, 2012; Cohen et al., 2018; Dagan et al., 2017; Khedr et al., 2020; Srovnalova et al., 2011) used rTMS multisite stimulation and nine studies (Chang et al., 2017; Cheng et al., 2022; W. He et al., 2021; Kim et al., 2015; Ma et al., 2019; Mi et al., 2019, 2020; Pal et al., 2010; Zhuang et al., 2020) used rTMS single‐site stimulation. Only four studies (Benninger et al., 2012; Cheng et al., 2022; Pal et al., 2010; Srovnalova et al, 2011) used an rTMS protocol with less than 1000 pulses per session, while the other 12 studies (Benninger et al., 2011; Chang et al., 2017; Cohen et al., 2018; Dagan et al., 2017; W. He et al., 2021; Khedr et al., 2020; Kim et al., 2015; Ma et al., 2019; Maruo et al., 2013; Mi et al., 2019, 2020; Zhuang et al., 2020) stimulated equal to or more than 1000 pulses per session. The total sessions of rTMS were between 1 and 24. Only one of the 16 studies used dual‐mode NIBS as a control measure (Chang et al., 2017), the rest of the studies used sham stimulation as a control. The follow‐up period ranged from immediately after rTMS to 90 days. The results from assessments conducted more than ten days following rTMS treatment were considered long‐term outcomes, otherwise they were considered short‐term results. Nearly all of the 16 studies conducted tests for outcome variables during the “on” state in PD. Only three studies reported 10‐m walking time (Benninger et al., 2011, 2012; Maruo et al., 2013), five studies (Chang et al., 2017; Cohen et al., 2018; Kim et al., 2015; Mi et al., 2020; Pal et al., 2010) performed timed up‐and‐go test (TUG), and seven studies (Benninger et al., 2012; Chang et al., 2017; Dagan et al., 2017; Kim et al., 2015; Ma et al., 2019; Mi et al., 2019, 2020) assessed freezing of gait questionnaire (FOG‐Q). In addition, cognitive function was assessed using Montreal cognitive assessment (MOCA) (Chang et al., 2017; Cheng et al., 2022; W. He et al., 2021; Khedr et al., 2020; Zhuang et al., 2020) and frontal assessment battery (FAB) (Benninger et al., 2011, 2012; Srovnalova et al., 2011).

### 3.3 Methodological quality of the included studies

The methodological quality of the included studies was assessed using the Cochrane Risk of Bias Assessment Tool. All 16 studies included in the meta‐analysis used random allocation, with 12 studies (Benninger et al., 2011, 2012; Chang et al., 2017; Cheng et al., 2022; Cohen et al., 2018; W. He et al., 2021; Khedr et al., 2020; Ma et al., 2019; Mi et al., 2019, 2020; Pal et al., 2010; Zhuang et al., 2020) using a randomized parallel design and four studies (Dagan et al., 2017; Kim et al., 2015; Maruo et al., 2013; Srovnalova et al., 2011) using a randomized crossover design. Sixteen studies reported information related to blinding, of which 14 (Benninger et al., 2011, 2012; Chang et al., 2017; Cohen et al., 2018; Dagan et al., 2017; W. He et al., 2021; Khedr et al., 2020; Kim et al., 2015; Ma et al., 2019; Maruo et al., 2013; Mi et al., 2019, 2020; Pal et al., 2010; Srovnalova et al., 2011) were double‐blind, and two (Cheng et al., 2022; Zhuang et al., 2020) were single‐blind. Only two studies (Cheng et al., 2022; Zhuang et al., 2020) reported that the outcome assessors were not blinded, and two other studies (Dagan et al., 2017; Srovnalova et al., 2011) did not mention whether the outcome assessors were blinded.

Eight studies (Benninger et al., 2011, 2012; Cheng et al., 2022; Cohen et al., 2018; Dagan et al., 2017; W. He et al., 2021; Khedr et al., 2020; Kim et al., 2015) documented the number of dropouts, while the remaining eight (Chang et al., 2017; Ma et al., 2019; Maruo et al., 2013; Mi et al., 2019, 2020; Pal et al., 2010; Srovnalova et al., 2011; Zhuang et al., 2020) did not report any dropouts. Selective reporting of findings was not found in all 16 studies. Furthermore, other sources of bias identified were the small sample sizes of the 16 studies included. A detailed evaluation of the methodological quality is provided in Figures S1 and S2.

### 3.4 Freezing of gait questionnaire

Compared to the control group, five studies (Benninger et al., 2012; Chang et al., 2017; Kim et al., 2015; Ma et al., 2019; Mi et al., 2020) with 150 participants (*n* = 84 in the treatment group and *n* = 66 in control) focused on how rTMS affected gait in patients with PD using FOG‐Q showed a statistically significant effect on post‐training assessment within ten days (WMD: −0.925,95% CI: −1.642 to −0.209, *p* = .011; Figure S3) with low heterogeneity (*χ* ^2^ = 3.220, *P* for *Q* statistic = .522, *I* ^2^ = 0.000%; Figure S3). The funnel plot did not indicate significant asymmetry (Figure S4). The formal statistical tests did not indicate publication bias in the five studies (Egger's test, *p* = .727).

Seven studies (Benninger et al., 2012; Chang et al., 2017; Dagan et al., 2017; Kim et al., 2015; Ma et al., 2019; Mi et al., 2019, 2020) with 194 participants (*n* = 111 in the treatment group and *n* = 83 in control) assessed the long‐term outcomes (assessed more than 10 days after training) of rTMS on gait using FOG‐Q. Long‐term efficacy of rTMS on the FOG‐Q was statistically significant (WMD: −2.120; 95% CI: −2.751 to −1.489, *p* = .000; *χ* ^2^ = 7.100, *P* for *Q* statistic = .312, *I* ^2 ^= 15.400%; Figure S5). No substantial asymmetry was observed in the funnel plot (Figure S6). Egger's test did not show publication bias for these seven studies (*p* = .072).

### 3.5 10‐m walking time

Using data from three studies (Benninger et al., 2011, 2012; Maruo et al., 2013) with 73 participants (*n* = 36 in the treatment group and *n* = 37 in the control group), we found that compared to sham rTMS, active rTMS improved the 10‐m walking time in the short term (WMD: −0.456, 95% CI: −0.793 to −0.119, *p* = .008; Figure S7). Heterogeneity between the included studies could be acceptable (*χ* ^2^ = 0.370, *P* for *Q* statistic = .829, *I* ^2 ^= 0.000%; Figure S7). There was no significant asymmetry in the funnel plot (Figure S8). Egger's test did not suggest publication bias for these three studies (*p* = .668).

The long‐term effect of rTMS on the 10‐m walking time was reported in two studies (Benninger et al., 2011, 2012) involving 52 participants [24, 26]. The long‐term effect on the 10 m walking time was evident after real rTMS (WMD: −0.526, 95% CI: −0.885 to −0.167, *p* = .004; Figure S9), and with a low level of heterogeneity in the estimates (*χ* ^2^ = 0.300, *P* for *Q* statistic = .582, *I* ^2 ^= 0.000%; Figure S9). The funnel plot showed low evidence of publication bias (Figure S10).

### 3.6 Timed up‐and‐go test

Four studies (Chang et al., 2017; Kim et al., 2015; Mi et al., 2020; Pal et al., 2010) with 118 participants (*n* = 65 in the treatment group and *n* = 53 in control) reported that the TUG was performed within 10 days after rTMS treatment. The findings indicated that patients with PD treated with real rTMS took significantly less time in the TUG trial than sham controls (WMD: −1.064, 95% CI: −1.555 to −0.572, *p* = .000; Figure S11). There was no significant heterogeneity in the included studies (*χ* ^2^ = 1.170, *P* for *Q* statistic = .761, *I* ^2 ^= 0.000%; Figure S11). The funnel plot was visually symmetrical. Egger's test did not suggest publication bias for these three studies (*p* = .146; Figure S12).

Furthermore, the long‐term outcomes of rTMS of 160 participants in five studies (Chang et al., 2017; Cohen et al., 2018; Kim et al., 2015; Mi et al., 2020; Pal et al., 2010) (86 in the treatment group and 74 in the control group) using the TUG test were assessed more than 10 days after training. The pooled data results showed that the time spent by patients with PD in the TUG trial was significantly shorter after the real rTMS therapy compared to the sham control (WMD: −1.097, 95% CI: −1.422 to −0.772, *p* = .000; Figure S13). Heterogeneity among the five included studies was acceptable (*χ* ^2^ = 1.160, *P* for *Q* statistic = .884, *I* ^2 ^= 0.000%; Figure S13). There was no significant asymmetry in the funnel plot (Figure S14). Egger's test did not indicate publication bias in these five studies (*p* = .179).

### 3.7 Montreal cognitive assessment

Five trials (Chang et al., 2017; Cheng et al., 2022; W. He et al., 2021; Khedr et al., 2020; Zhuang et al., 2020) with 160 participants (*n* = 84 in the treatment group and *n* = 76 in the control group) evaluated the efficacy of rTMS on global cognition using MOCA. There was a significant difference between the active rTMS and control group (WMD: 2.670, 95% CI: 0.513 to 4.827, *p* = .015; Figure S15). The heterogeneity of the included studies exceed the expected contingency (*I* ^2 ^= 75.900%, *χ* ^2^ = 16.590, *P* for *Q* statistic = .002; Figure S15), suggesting that the results of the included studies exhibited substantial heterogeneity. The sensitivity analysis results (Figure S16) of the trials indicated that after removing the highly weighted study (Chang et al., 2017), the *I* ^2^ statistics between the studies was 0.00% (*χ* ^2^ = 1.770, *P* for *Q* statistic = .622; Figure S17). Moreover, the differences between the two groups were still significant (WMD: 3.714, 95% CI: 2.567 to 4.861, *p* = .000; Figure S17). The funnel plot of the four included studies revealed symmetrical distribution (Figure S18). The Egger's test reported insignificant results for publication bias (*p* = .927).

### 3.8 Frontal assessment battery

Three studies (Benninger et al., 2011, 2012; Srovnalova et al., 2011), which included 72 subjects (36 in the treatment group and 36 in the control group), evaluated the efficacy of rTMS on the FAB. The pooled statistics revealed a positive effect in favor of rTMS (WMD: −0.584, 95% CI: −0.934 to −0.234, *p* = .001; Figure S19) on FAB without heterogeneity (*χ* ^2^ = 2.630, *P* for *Q* statistic = .2680, *I* ^2 ^= 24.000%; Figure S19). The funnel plot of the included studies was symmetrically distributed (Figure S20), and Egger's test did not suggest publication bias (*p* = .427).

## 4 DISCUSSION

This updated meta‐analysis was conducted to further assess the efficacy of rTMS on gait and cognition in patients with PD. Based on the results of this meta‐analysis, we can draw several conclusions. First, our meta‐analysis found that rTMS was effective in improving the FOG‐Q scores, 10‐m walking time, TUG scores, MOCA, and FAB. Almost all measurements were performed in the “on” state in PD. Second, this study found that rTMS produced short‐term and long‐term effects on FOG‐Q, 10‐m walking time, and TUG outcomes in patients with PD in the subgroup analysis. Third, the rTMS stimulation protocols used in the included studies in this meta‐analysis were diverse and heterogeneous. Of these, most studies (93.750%) used rTMS protocols with high‐frequency (> 1 Hz) stimulation. Moreover, 56.250% of the studies used rTMS single‐site stimulation protocols, while 43.750% used rTMS multi‐site stimulation. The primary motor cortex (M1), prefrontal cortex (PFC), and the SMA were the most common rTMS stimulation sites. Only 25% of the studies used rTMS protocols with less than 1000 pulses per session, while 75% of the studies were equal to or more than 1000 pulses per session.

This meta‐analysis revealed the beneficial effect of rTMS on the FOG‐Q and TUG scores in the short‐ and long term. This is inconsistent with the previous meta‐analysis by Xie et al. (2020), which showed no significant differences between the active rTMS and control group in the FOG‐Q and TUG scores with high heterogeneity. These distinctions may be due to differences in the included trials, data extraction, and statistical methods. Compared with the previously published meta‐analysis, this study was analyzed in subgroups according to whether the follow‐up period was longer than 10 days. In addition, we used WMD as the effect size for the meta‐analysis, while Xie used standardized mean difference (SMD). Generally speaking, WMD eliminates the effect of absolute magnitude on the results (Faraone, 2008) so that the original metric of the study truly reflects the effect of the test. However, SMD eliminates the effect of absolute magnitude and the effect of the metric on the outcome (Faraone, 2008) so that SMD becomes a unitless value that can only account for statistical significance and cannot be interpreted well in the context of clinical significance. Our findings suggest that active rTMS can reduce walking time in patients with PD compared to sham rTMS, which is consistent with previous reviews by Chung and Mak (2016) and Kim et al. (2019). Compared to our study, Chung's study included a larger number of studies that assessed gait speed in various ways that were not limited to the 10 m walking time. However, Chung and Mak's (2016) study reported significant publication bias results and high heterogeneity. In Kim et al.’s (2019) meta‐analysis, the study population was not purely PD, and the intervention included transcranial direct current stimulation in addition to rTMS.

In line with a previous meta‐analysis by Jiang et al. (2020), our overall pooled results suggest that rTMS has a positive impact on cognitive function in PD. Jiang et al. (2020) used the mini‐mental state examination to observe the effect of rTMS on global cognitive function, whereas we used the MOCA outcome. On the other hand, the meta‐analysis by Jiang et al. (2020) included 11 studies and used a variety of outcomes such as the executive function index and FAB. To assess the effect of rTMS on executive function, we only included three studies in which FAB was selected as the outcome. However, a meta‐analysis by P. K. He et al. (2020) came to a different conclusion suggesting that rTMS failed to improve global cognition, executive function, attention, and working memory in PD. The primary reasons for these differences may be variations in the included studies and statistical effect sizes. In contrast to the study of P. K. He et al. (2020), we excluded one Korean language study (Oh et al., 2015) and another original study (Randver et al., 2019) on PD combined with refractory depression in fewer than 10 subjects to reduce the sources of bias. In addition, for the MOCA outcome, we included two recently published studies (Cheng et al., 2022; W. He et al., 2021) in our analysis.

Concerning the stimulation protocols, our study suggested that rTMS stimulus lacks a uniform paradigm. Unfortunately, due to limitations in the number of original studies, we could not perform subgroup analyses of certain important parameters of rTMS. We speculated from the data extraction results that the choice of rTMS stimulation protocol might be superior for improving FOG and cognitive function, which used high frequencies (> 1 Hz) and equal to or more than 1000 pulses per session in the M1, PFC, and SMA. A previous meta‐analysis by Yang et al. (2018) also found that the high‐frequency rTMS therapy over the M1 with a total of 18,000–20,000 pulses appears to have better efficacy on the motor signs of PD. The meta‐analysis by Jiang et al. (2020) showed that multiple sessions of high‐frequency rTMS over the DLPFC may have a positive effect on executive function in patients with PD. In summary, large, well‐designed randomized controlled trials will be needed in the future to confirm these speculations.

The pathogenesis of FOG is unclear and considered to be related to several brain areas and neural circuits (Jha et al., 2015). A study (Jha et al., 2015) that used a voxel‐based morphometry analysis showed significant gray matter atrophy in the FOG group in the left temporal and right frontal areas as well as significant involvement of the right cerebellum. Recent research has suggested that cognitive impairment plays a role in the formation of FOG (Dagan et al., 2017). Its close connection with cognitive disorders has been proposed, and some researchers explain the pathogenesis using the cognitive model theory (Vandenbossche et al., 2012). This review (Vandenbossche et al., 2012) proposes that as both automatic and controlled processes are more severely damaged in freezers, the cognitive compensation in FOG is hindered, leading to potential gait disorder. Therefore, we hypothesize that if rTMS can work by improving cognitive function, then FOG may be alleviated simultaneously.

Although our results showed improvements in gait and cognitive function in PD, several limitations need to be acknowledged. First, our results may be constrained by the unclear risk of bias on certain domains due to incomplete data from some studies. Second, the total number of included studies and participants was small and may not be sufficient to explain individual differences. We only included studies of patients with primary PD, and these studies used assessment methods that were relatively specific to gait and cognitive function. However, there are currently few such RCT studies, and more high‐quality RCT studies are needed in the future. Second, the presence of some uncontrolled variables may confound the results, such as the baseline clinical data of patients with PD and the stimulation protocol of rTMS. To draw accurate conclusions about the effectiveness of rTMS in improving motor and cognitive symptoms in patients with PD, future studies need to standardize clinical information such as age, sex, medication use, H–Y staging, side of onset, and rTMS stimulation site, frequency, and intensity.

## 5 CONCLUSION

In conclusion, our meta‐analysis suggests that rTMS is a feasible technique to improve FOG and certain cognitive domains in patients with PD. Unfortunately, due to the limited number of studies, no subgroup analysis of the rTMS stimulation parameters could be conducted to assess the effects of different stimulation parameters on the motor and cognitive outcomes. To be able to translate rTMS into a viable form of clinical treatment, a better understanding of how different rTMS parameters affect motor and cognitive function is necessary to induce optimal improvements in the functioning of patients with PD.

## CONFLICT OF INTEREST

The authors declare that they have no conflict of interest.

### PEER REVIEW

The peer review history for this article is available at https://publons.com/publon/10.1002/brb3.2697

## Supporting information

Supplementary Information

Supplementary Information

## Data Availability

The original contributions presented in the study are included in the article/supplementary material, further inquiries can be directed to the corresponding author.
