# Supplementary material for: Effects of repetitive transcranial magnetic stimulation on gait disorders and cognitive dysfunction in Parkinson's disease: A systematic review with meta‐analysis
Source: Brain Behav. 2022 Jul 21;12(8):e2697. doi: 10.1002/brb3.2697 (PMC9392523; doi:10.1002/brb3.2697)
Supplement: Supplementary file 1 — Supplementary Information [file BRB3-12-e2697-s001.docx]

**Table S1.** Search strategies

| Database |  | Step | Search algorithm |
| --- | --- | --- | --- |
| Pubmed |  | 1 | ((((((((((((((((((Idiopathic Parkinson's Disease[Title/Abstract])) OR (Lewy Body Parkinson's Disease[Title/Abstract])) OR (Parkinson's Disease, Idiopathic[Title/Abstract])) OR (Parkinson's Disease, Lewy Body[Title/Abstract])) OR (Parkinson Disease, Idiopathic[Title/Abstract])) OR (Parkinson's Disease[Title/Abstract])) OR (Idiopathic Parkinson Disease[Title/Abstract])) OR (Lewy Body Parkinson Disease[Title/Abstract])) OR (Primary Parkinsonism[Title/Abstract])) OR (Parkinsonism, Primary[Title/Abstract])) OR (Paralysis Agitans[Title/Abstract])) OR (PD[Title/Abstract])) OR "Parkinson Disease"[Mesh]) |
|  |  | 2 | (() AND Gait Disorders, Neurologic[Mesh]) OR ((((((((((((((((((((((((((((Neurologic Gait Disorder[Title/Abstract])) OR (Neurologic Gait Disorders[Title/Abstract])) OR (Neurologic Locomotion Disorders[Title/Abstract])) OR (Locomotion Disorders, Neurologic[Title/Abstract])) OR (Locomotion Disorder, Neurologic[Title/Abstract])) OR (Neurologic Locomotion Disorder[Title/Abstract])) OR (Neurologic Ambulation Disorders[Title/Abstract])) OR (Ambulation Disorders, Neurologic[Title/Abstract])) OR (Ambulation Disorder, Neurologic[Title/Abstract])) OR (Neurologic Ambulation Disorder[Title/Abstract])) OR (Gait Dysfunction, Neurologic[Title/Abstract])) OR (Gait Dysfunctions, Neurologic[Title/Abstract])) OR (Neurologic Gait Dysfunction[Title/Abstract])) OR (Neurologic Gait Dysfunctions[Title/Abstract])) OR (Gait, Festinating[Title/Abstract])) OR (Gait, Rigid[Title/Abstract])) OR (Rigid Gait[Title/Abstract])) OR (Gait, Spastic[Title/Abstract])) OR (Spastic Gait[Title/Abstract])) OR (motor function[Title/Abstract])) OR (FOG[Title/Abstract])) OR (The gait disorder[Title/Abstract])) OR (gait disturbance[Title/Abstract])) OR (freezing of gait[Title/Abstract])) OR (gait impairments[Title/Abstract])) OR (Motor Symptoms[Title/Abstract])) OR (motor disturbance[Title/Abstract])) OR (locomotor function[Title/Abstract])) |
|  |  | 3 | ( "Cognitive Dysfunction"[Mesh]) OR ((((((((((((((((((((((((Cognition Disorders[Title/Abstract])) OR (Cognitive Dysfunctions[Title/Abstract])) OR (Dysfunction, Cognitive[Title/Abstract])) OR (Dysfunctions, Cognitive[Title/Abstract])) OR (Cognitive Impairments[Title/Abstract])) OR (Cognitive Impairment[Title/Abstract])) OR (Impairment, Cognitive[Title/Abstract])) OR (Impairments, Cognitive[Title/Abstract])) OR (Mild Cognitive Impairment[Title/Abstract])) OR (Cognitive Impairment, Mild[Title/Abstract])) OR (Cognitive Impairments, Mild[Title/Abstract])) OR (Impairment, Mild Cognitive[Title/Abstract])) OR (Impairments, Mild Cognitive[Title/Abstract])) OR (Mild Cognitive Impairments[Title/Abstract])) OR (Mild Neurocognitive Disorder[Title/Abstract])) OR (Disorder, Mild Neurocognitive[Title/Abstract])) OR (Disorders, Mild Neurocognitive[Title/Abstract])) OR (Mild Neurocognitive Disorders[Title/Abstract])) OR (Neurocognitive Disorder, Mild[Title/Abstract])) OR (Neurocognitive Disorders, Mild[Title/Abstract])) OR (Cognitive Decline[Title/Abstract])) OR (Cognitive Declines[Title/Abstract])) OR (Decline, Cognitive[Title/Abstract])) OR (Declines, Cognitive[Title/Abstract])) |
|  |  | 4 | ("Dementia"[Mesh]) OR ((((Dementias[Title/Abstract])) OR (MCI[Title/Abstract])) OR (PDD[Title/Abstract])) OR (Parkinson's disease dementia[Title/Abstract])) |
|  |  | 5 | ((((((((((Magnetic Stimulation, Transcranial[Title/Abstract])) OR (Magnetic Stimulations, Transcranial[Title/Abstract])) OR (Stimulation, Transcranial Magnetic[Title/Abstract])) OR (Stimulations, Transcranial Magnetic[Title/Abstract])) OR (Transcranial Magnetic Stimulations[Title/Abstract])) OR (Transcranial Magnetic Stimulation, Single Pulse[Title/Abstract])) OR (Transcranial Magnetic Stimulation, Paired Pulse[Title/Abstract])) OR (Transcranial Magnetic Stimulation, Repetitive[Title/Abstract])) OR (rTMS[Title/Abstract])) OR "Transcranial Magnetic Stimulation"[Mesh]) |
|  |  | 6 | ((randomizedcontrolledtrial[Filter]) |
|  |  | 7 | (humans[Filter])) |
|  |  | 8 | 1 AND 2 AND 3 AND 4 AND 5 AND 6 AND 7 |
| Embass |  | #1 | MeSH descriptor:Parkinson Disease |
|  |  | #2 | 'Idiopathic Parkinson Disease':ab,ti OR 'Lewy Body Parkinson Disease':ab,ti OR 'Parkinson Disease, Idiopathic':ab,ti OR 'Parkinson Disease, Lewy Body':ab,ti OR 'Parkinson Disease, Idiopathic':ab,ti OR 'Parkinson Disease':ab,ti OR 'Idiopathic Parkinson Disease':ab,ti OR 'Lewy Body Parkinson Disease':ab,ti OR 'Primary Parkinsonism':ab,ti OR 'Parkinsonism, Primary':ab,ti OR 'Paralysis Agitans':ab,ti OR 'PD':ab,ti |
|  |  | #3 | #1 OR #2 |
|  |  | #4 | MeSH descriptor:Gait Disorders, Neurologic |
|  |  | #5 | 'Gait Disorder, Neurologic':ab,ti OR 'Neurologic Gait Disorder':ab,ti OR 'Neurologic Gait Disorders':ab,ti OR 'Neurologic Locomotion Disorders':ab,ti OR 'Locomotion Disorders, Neurologic':ab,ti OR 'Locomotion Disorder, Neurologic':ab,ti OR 'Neurologic Locomotion Disorder':ab,ti OR 'Neurologic Ambulation Disorders':ab,ti OR 'Ambulation Disorders, Neurologic':ab,ti OR 'Ambulation Disorder, Neurologic':ab,ti OR 'Neurologic Ambulation Disorder':ab,ti OR 'Gait Dysfunction, Neurologic':ab,ti OR 'Gait Dysfunctions, Neurologic':ab,ti OR 'Neurologic Gait Dysfunction':ab,ti OR 'Neurologic Gait Dysfunctions':ab,ti OR 'Gait, Festinating':ab,ti OR 'Gait, Rigid':ab,ti OR 'Rigid Gait':ab,ti OR 'Gait, Spastic':ab,ti OR 'Spastic Gait':ab,ti OR 'motor function':ab,ti OR 'FOG':ab,ti OR 'The gait disorder':ab,ti OR 'The gait disorder':ab,ti OR 'freezing of gait':ab,ti OR 'gait disorders':ab,ti OR 'gait impairments':ab,ti OR 'Motor Symptoms':ab,ti OR 'motor disturbance':ab,ti OR 'locomotor function':ab,ti |
|  |  | #6 | #4 OR #5 |
|  |  | #7 | MeSH descriptor:Cognitive Dysfunction |
|  |  | #8 | 'Cognition Disorders':ab,ti OR 'Cognitive Dysfunctions':ab,ti OR 'Dysfunction, Cognitive':ab,ti OR 'Dysfunctions, Cognitive':ab,ti OR 'Cognitive Impairments':ab,ti OR 'Cognitive Impairment':ab,ti OR 'Impairment, Cognitive':ab,ti OR 'Impairments, Cognitive':ab,ti OR 'Mild Cognitive Impairment':ab,ti OR 'Cognitive Impairment, Mild':ab,ti OR 'Cognitive Impairments, Mild':ab,ti OR 'Impairment, Mild Cognitive':ab,ti OR 'Impairments, Mild Cognitive':ab,ti OR 'Mild Cognitive Impairments':ab,ti OR 'Mild Neurocognitive Disorder':ab,ti OR 'Disorder, Mild Neurocognitive':ab,ti OR 'Disorders, Mild Neurocognitive':ab,ti OR 'Mild Neurocognitive Disorders':ab,ti OR 'Neurocognitive Disorder, Mild':ab,ti OR 'Neurocognitive Disorders, Mild':ab,ti OR 'Cognitive Decline':ab,ti OR 'Cognitive Declines':ab,ti OR 'Decline, Cognitive':ab,ti OR 'Declines, Cognitive':ab,ti OR 'dementia':ab,ti OR 'Dementias':ab,ti OR 'MCI':ab,ti OR 'PDD':ab,ti OR 'Parkinson disease dementia':ab,ti |
|  |  | #9 | #7 OR #8 |
|  |  | #10 | MeSH descriptor:Transcranial Magnetic Stimulation |
|  |  | #11 | 'Magnetic Stimulation, Transcranial':ab,ti OR 'Magnetic Stimulations, Transcranial':ab,ti OR 'Stimulation, Transcranial Magnetic':ab,ti OR 'Stimulations, Transcranial Magnetic':ab,ti OR 'Transcranial Magnetic Stimulations':ab,ti OR 'Transcranial Magnetic Stimulation, Single Pulse':ab,ti OR 'Transcranial Magnetic Stimulation, Paired Pulse':ab,ti OR 'Transcranial Magnetic Stimulation, Repetitive':ab,ti OR ' rTMS':ab,ti |
|  |  | #12 | #10 OR #11 |
|  |  | #13 | 'randomized controlled trial':ab,ti OR 'randomized':ab,ti OR 'placebo':ab,ti OR 'RCT':ab,ti |
|  |  | #14 | #3 AND #6 AND #9 AND #12 AND #13 |
| Cochrane |  | #1 | MeSH descriptor:Parkinson Disease |
|  |  | #2 | (Idiopathic Parkinson's Disease):ab,ti,kw or (Lewy Body Parkinson's Disease):ab,ti,kw or (Parkinson's Disease, Idiopathic):ab,ti,kw or (Parkinson's Disease, Lewy Body):ab,ti,kw or (Parkinson Disease, Idiopathic):ab,ti,kw or (Parkinson's Disease):ab,ti,kw or (Idiopathic Parkinson Disease):ab,ti,kw or (Lewy Body Parkinson Disease):ab,ti,kw or (Primary Parkinsonism):ab,ti,kw or (Parkinsonism, Primary):ab,ti,kw or (Paralysis Agitans):ab,ti,kw or (PD):ab,ti,kw |
|  |  | #3  #4 | #1 OR #2  MeSH descriptor: Gait Disorders, Neurologic |
|  |  | #5 | (Gait Disorder, Neurologic):ab,ti,kw OR (Neurologic Gait Disorder):ab,ti,kw OR (Neurologic Gait Disorders):ab,ti,kw OR (Neurologic Locomotion Disorders):ab,ti,kw OR (Locomotion Disorders, Neurologic):ab,ti,kw OR (Locomotion Disorder, Neurologic):ab,ti,kw OR (Neurologic Locomotion Disorder):ab,ti,kw OR (Neurologic Ambulation Disorders):ab,ti,kw OR (Ambulation Disorders, Neurologic):ab,ti,kw OR (Ambulation Disorder, Neurologic):ab,ti,kw OR (Neurologic Ambulation Disorder):ab,ti,kw OR (Gait Dysfunction, Neurologic):ab,ti,kw OR (Gait Dysfunctions, Neurologic):ab,ti,kw OR (Neurologic Gait Dysfunction):ab,ti,kw OR (Neurologic Gait Dysfunctions):ab,ti,kw OR (Gait, Festinating):ab,ti,kw OR (Gait, Rigid):ab,ti,kw OR (Rigid Gait):ab,ti,kw OR (Gait, Spastic):ab,ti,kw OR (Spastic Gait):ab,ti,kw OR (motor function):ab,ti,kw OR (FOG):ab,ti,kw OR (The gait disorder):ab,ti,kw OR (The gait disorder):ab,ti,kw OR (freezing of gait):ab,ti,kw OR (gait disorders):ab,ti,kw OR (gait impairments):ab,ti,kw OR (Motor Symptoms):ab,ti,kw OR (motor disturbance):ab,ti,kw OR (locomotor function):ab,ti,kw |
|  |  | #6  #7 | #4 OR #5  MeSH descriptor:Cognitive Dysfunction |
|  |  | #8  #9 | (Cognition Disorders):ab,ti,kw OR (Cognitive Dysfunctions):ab,ti,kw OR (Dysfunction, Cognitive):ab,ti,kw OR (Dysfunctions, Cognitive):ab,ti,kw OR (Cognitive Impairments):ab,ti,kw OR (Cognitive Impairment):ab,ti,kw OR (Impairment, Cognitive):ab,ti,kw OR (Impairments, Cognitive):ab,ti,kw OR (Mild Cognitive Impairment):ab,ti,kw OR (Cognitive Impairment, Mild):ab,ti,kw OR (Cognitive Impairments, Mild):ab,ti,kw OR (Impairment, Mild Cognitive):ab,ti,kw OR (Impairments, Mild Cognitive):ab,ti,kw OR (Mild Cognitive Impairments):ab,ti,kw OR (Mild Neurocognitive Disorder):ab,ti,kw OR (Disorder, Mild Neurocognitive):ab,ti,kw OR (Disorders, Mild Neurocognitive):ab,ti,kw OR (Mild Neurocognitive Disorders):ab,ti,kw OR (Neurocognitive Disorder, Mild):ab,ti,kw OR (Neurocognitive Disorders, Mild):ab,ti,kw OR (Cognitive Decline):ab,ti,kw OR (Cognitive Declines):ab,ti,kw OR (Decline, Cognitive):ab,ti,kw OR (Declines, Cognitive):ab,ti,kw OR (dementia):ab,ti,kw OR (Dementias):ab,ti,kw OR (MCI):ab,ti,kw OR (PDD):ab,ti,kw OR (Parkinson's disease dementia):ab,ti,kw  #7 OR #8 |
|  |  | #10 | MeSH descriptor:Transcranial Magnetic Stimulation |
|  |  | #11 | (Magnetic Stimulation, Transcranial):ab,ti,kw OR (Magnetic Stimulations, Transcranial):ab,ti,kw OR (Stimulation, Transcranial Magnetic):ab,ti,kw OR (Stimulations, Transcranial Magnetic):ab,ti,kw OR (Transcranial Magnetic Stimulations):ab,ti,kw OR (Transcranial Magnetic Stimulation, Single Pulse):ab,ti,kw OR (Transcranial Magnetic Stimulation, Paired Pulse):ab,ti,kw OR (Transcranial Magnetic Stimulation, Repetitive):ab,ti,kw OR ( rTMS):ab,ti,kw |
|  |  | #12  #13 | #10 OR #11  MeSH descriptor:randomized controlled trial |
|  |  | #14 | (randomized):ab,ti,kw OR (placebo):ab,ti,kw OR (RCT):ab,ti,kw |
|  |  | #15 | #13 OR #14 |
|  |  | #16 | #3 AND #6AND #9AND #12 AND #15 |
