# Supplementary material for: Effects of repetitive transcranial magnetic stimulation on gait disorders and cognitive dysfunction in Parkinson's disease: A systematic review with meta‐analysis
Source: Brain Behav. 2022 Jul 21;12(8):e2697. doi: 10.1002/brb3.2697 (PMC9392523; doi:10.1002/brb3.2697)
Supplement: Supplementary file 2 — Supplementary Information [file BRB3-12-e2697-s002.docx]

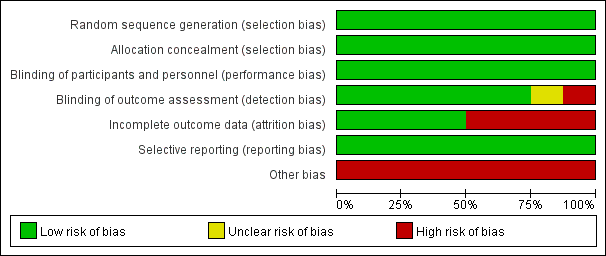


**Supplementary Figure S1.** Results of risk of bias assessment for individual level studies


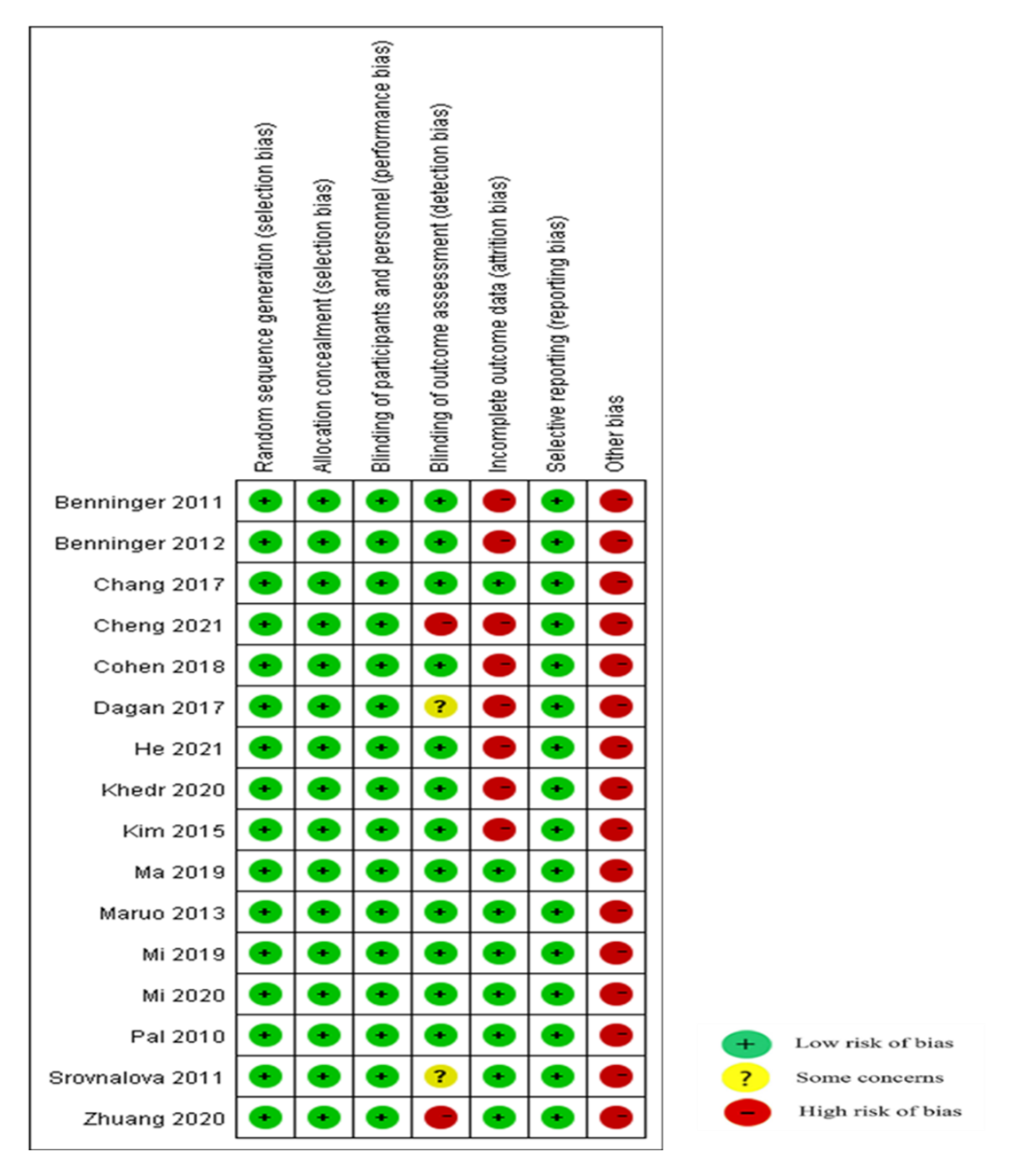


**Supplementary Figure S2.** An overall summary of the risk of bias assessment for the included studies


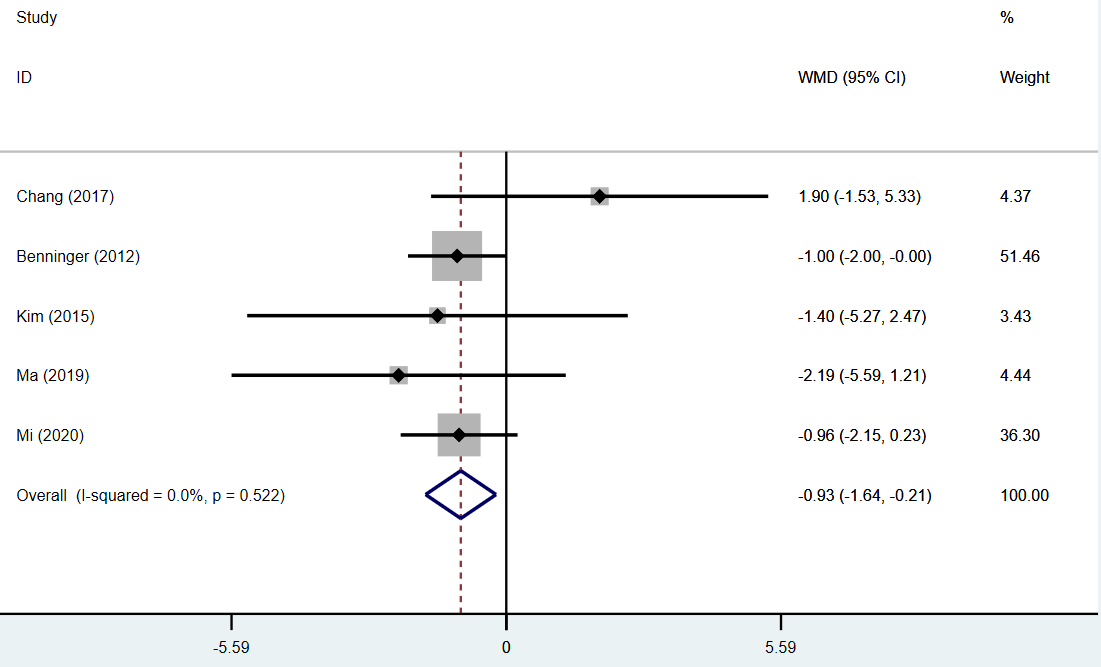


**Supplementary Figure S3.** Forest plot for the short-term effect of repetitive transcranial magnetic stimulation on Freezing of Gait Questionnaire


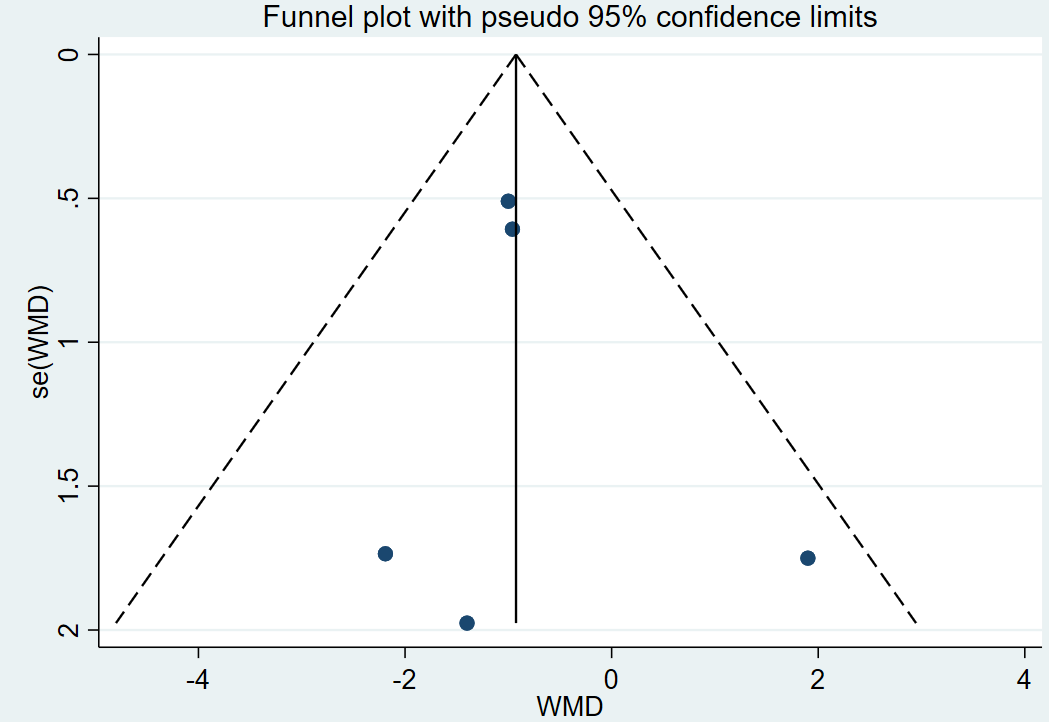


**Supplementary Figure S4.** Funnel plot for the short-term effect of repetitive transcranial magnetic stimulation on Freezing of Gait Questionnaire


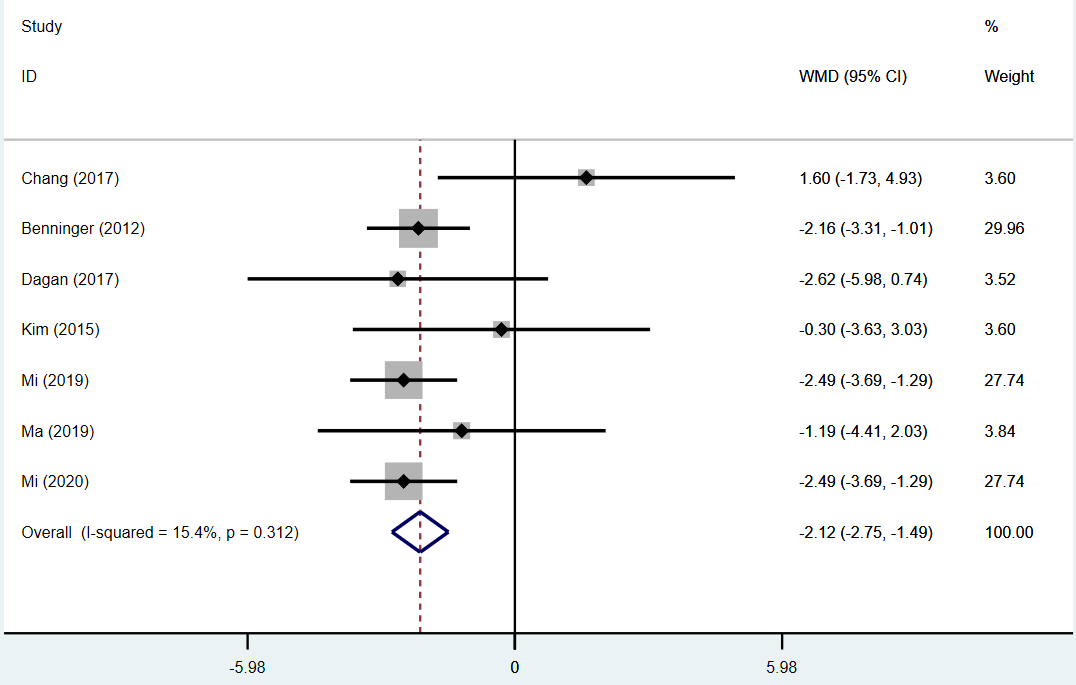


**Supplementary Figure S5.** Forest plot for the long-term effect of repetitive transcranial magnetic stimulation on Freezing of Gait Questionnaire


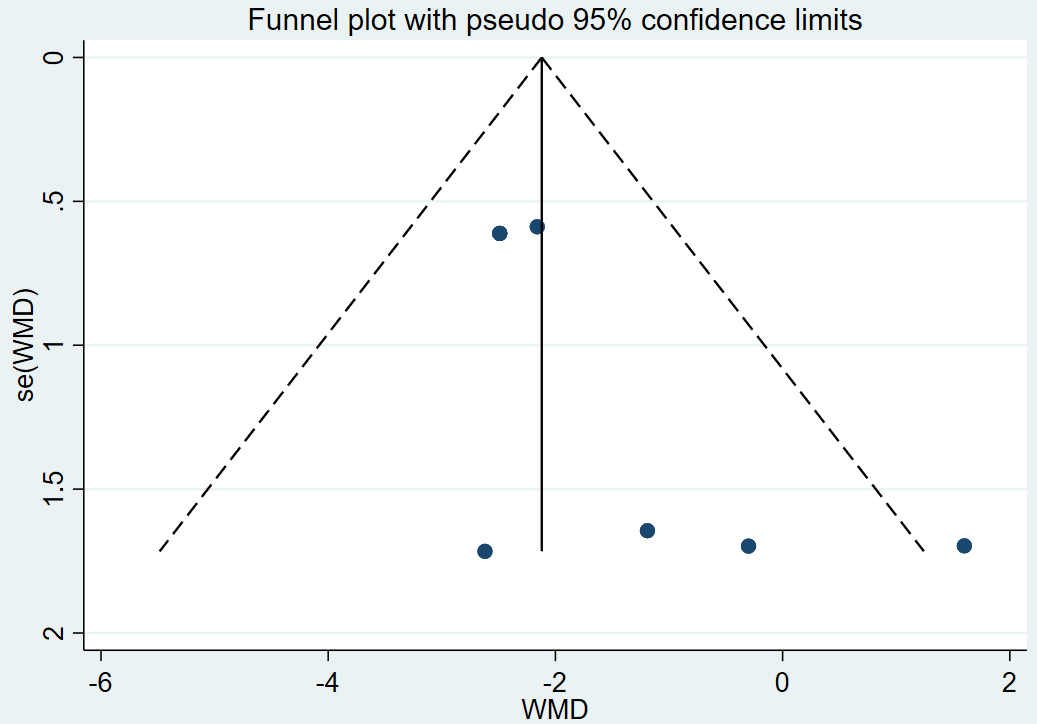


**Supplementary Figure S6.** Funnel plot for the long-term effect of repetitive transcranial magnetic stimulation on Freezing of Gait Questionnaire

**
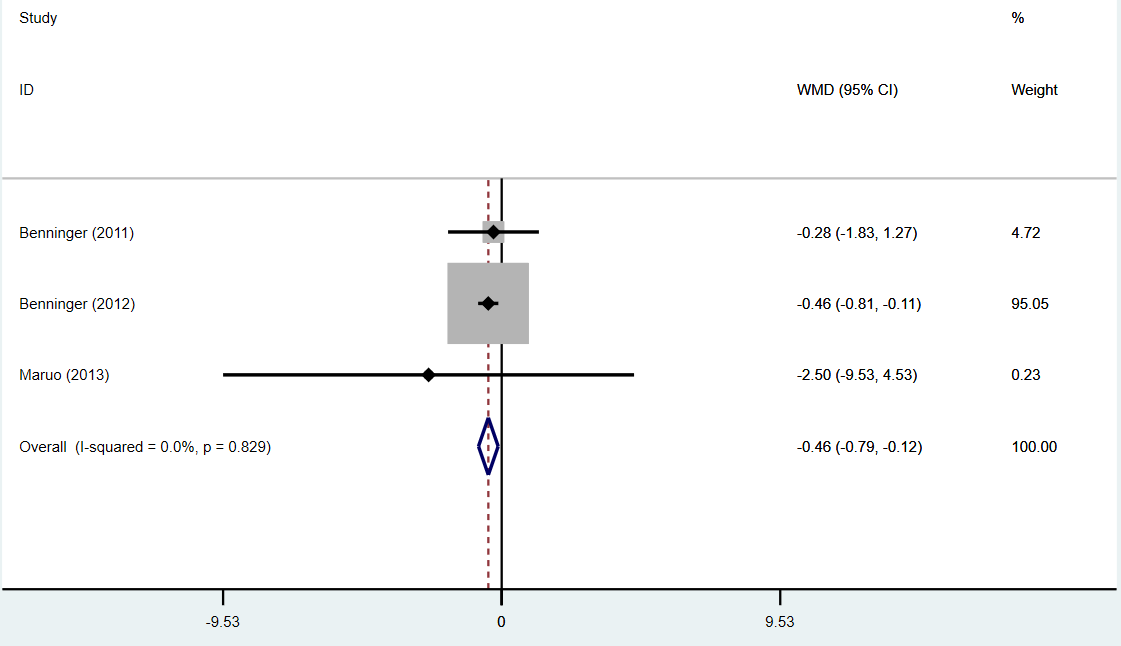
**

**Supplementary Figure S7.** Forest plot for the short-term effect of repetitive transcranial magnetic stimulation on 10-m walking time

**
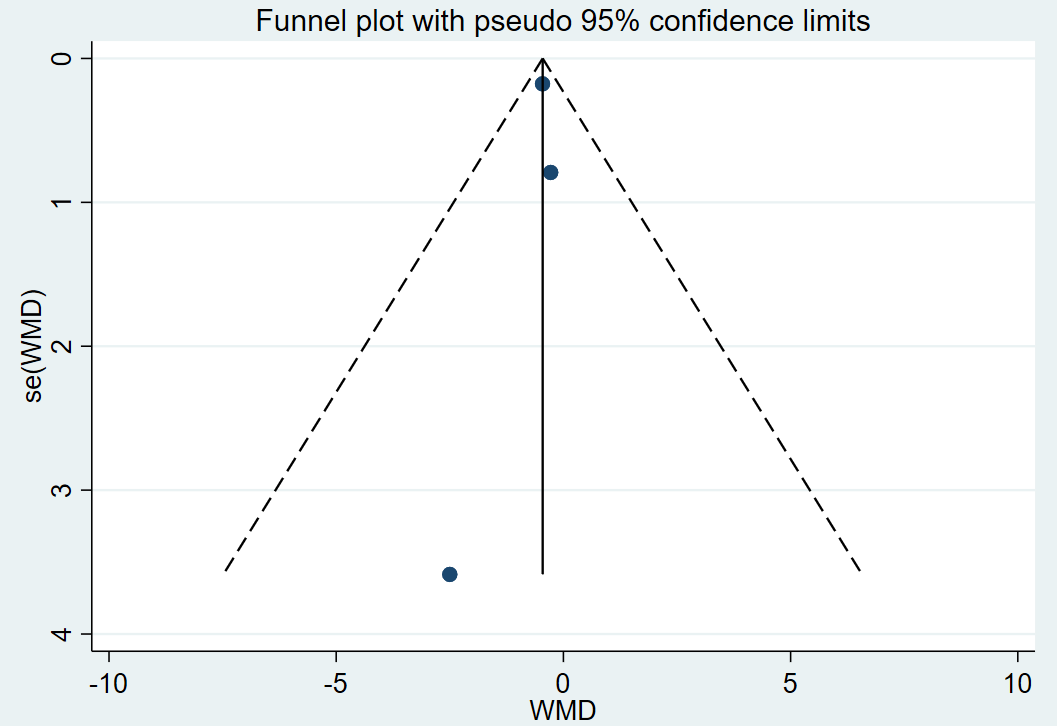
**

**Supplementary Figure S8.** Funnel plot for the short-term effect of repetitive transcranial magnetic stimulation on 10-m walking time

**
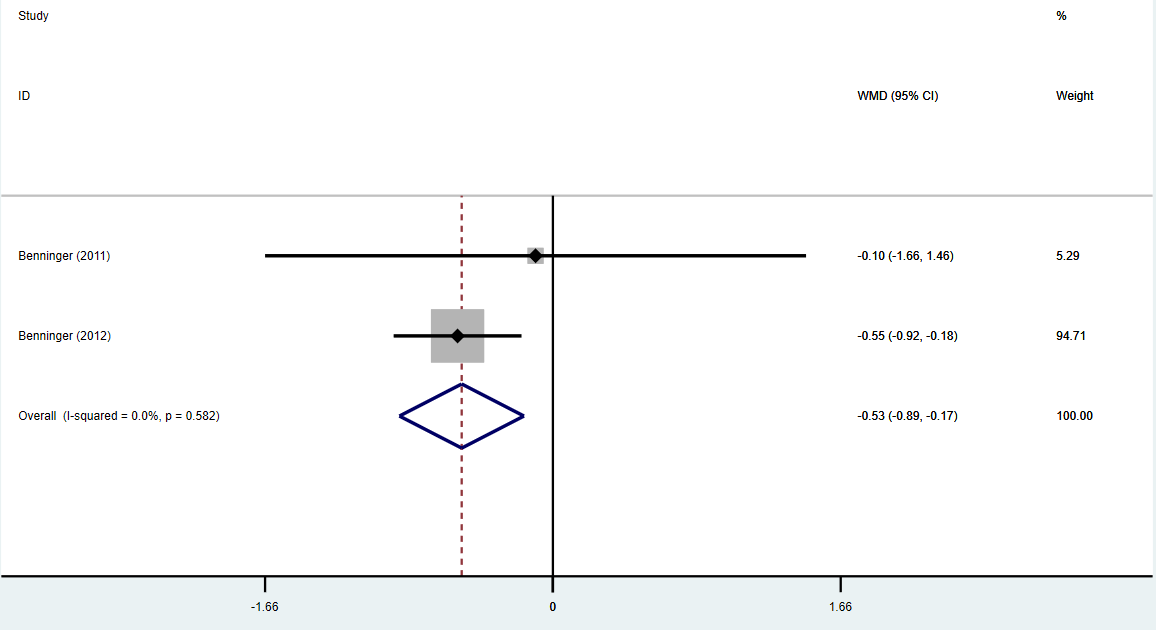
**

**Supplementary Figure S9.** Forest plot for the long-term effect of repetitive transcranial magnetic stimulation on 10-m walking time

**
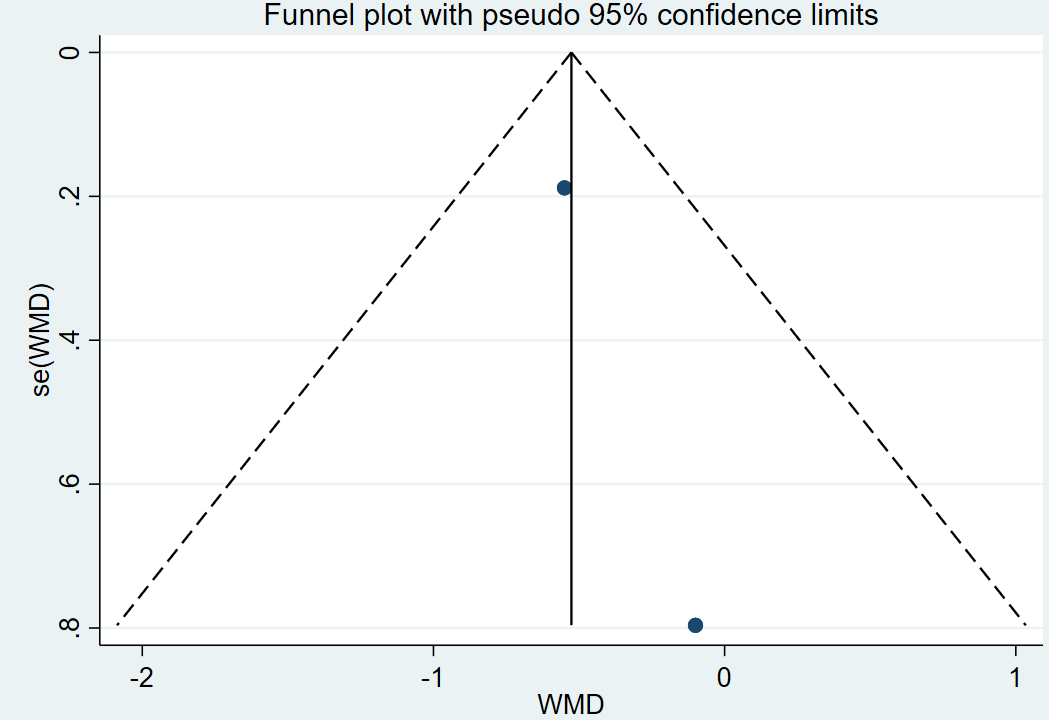
**

**Supplementary Figure S10.** Funnel plot for the long-term effect of repetitive transcranial magnetic stimulation on 10-m walking time

**
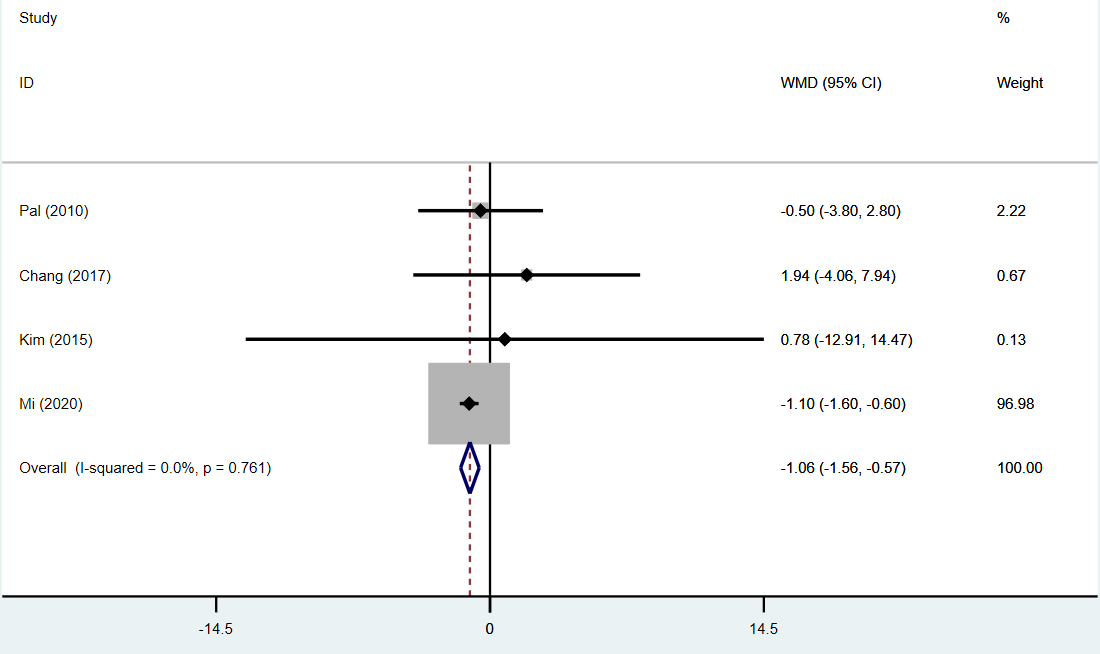
**

**Supplementary Figure S11.** Forest plot for the short-term effect of repetitive transcranial magnetic stimulation on Timed Up-and-Go test

**
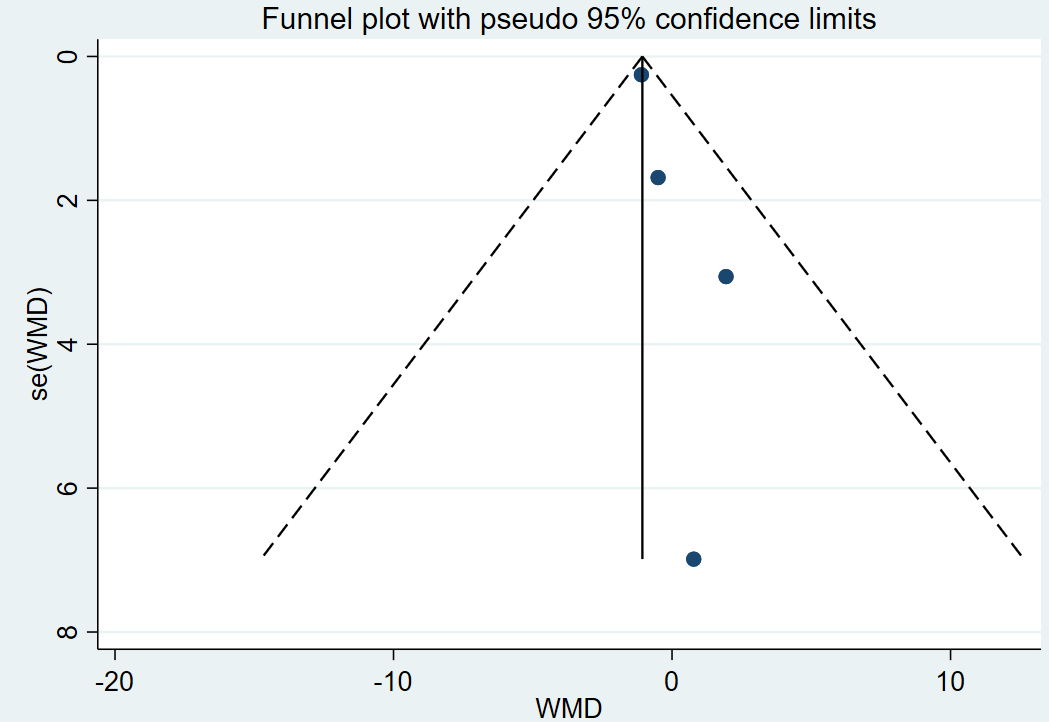
**

**Supplementary Figure S12.** Funnel plot for the short-term effect of repetitive transcranial magnetic stimulation on Timed Up-and-Go test

**
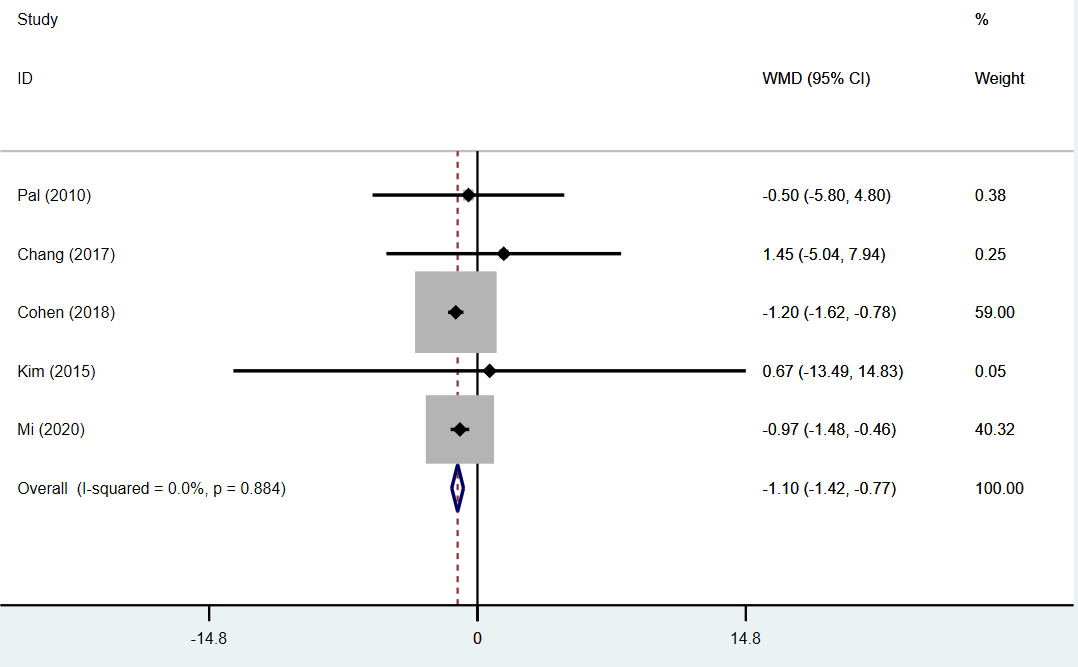
**

**Supplementary Figure S13.** Forest plot for the long-term effect of repetitive transcranial magnetic stimulation on Timed Up-and-Go test

**
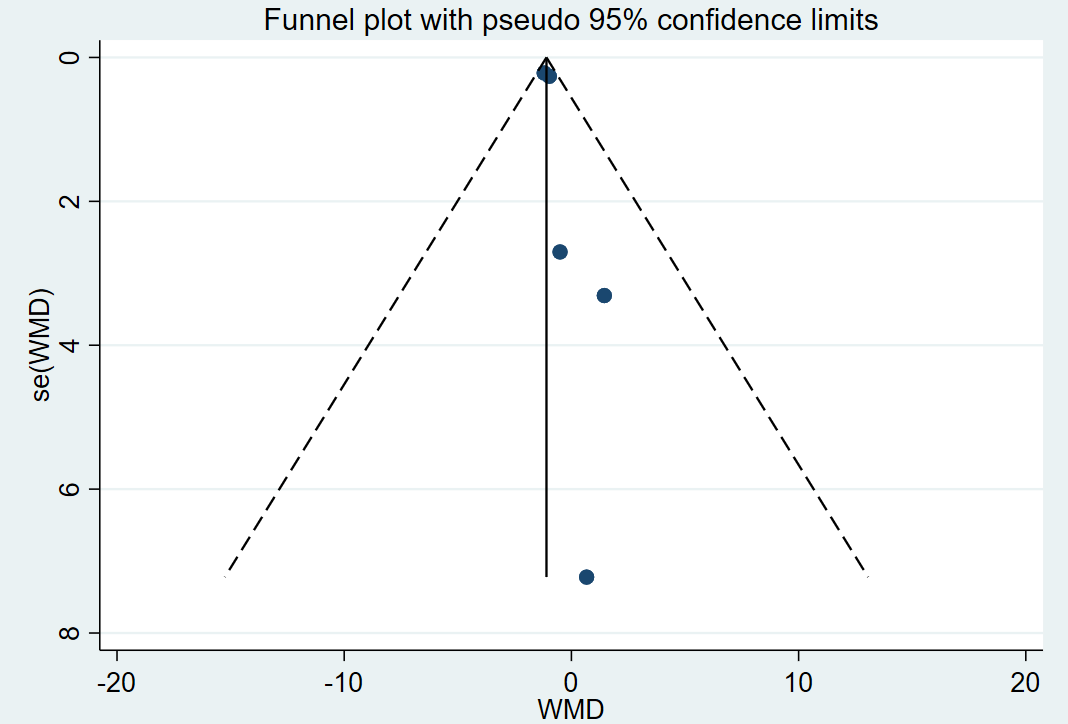
**

**Supplementary Figure S14.** Funnel plot for the long-term effect of repetitive transcranial magnetic stimulation on Timed Up-and-Go test

**
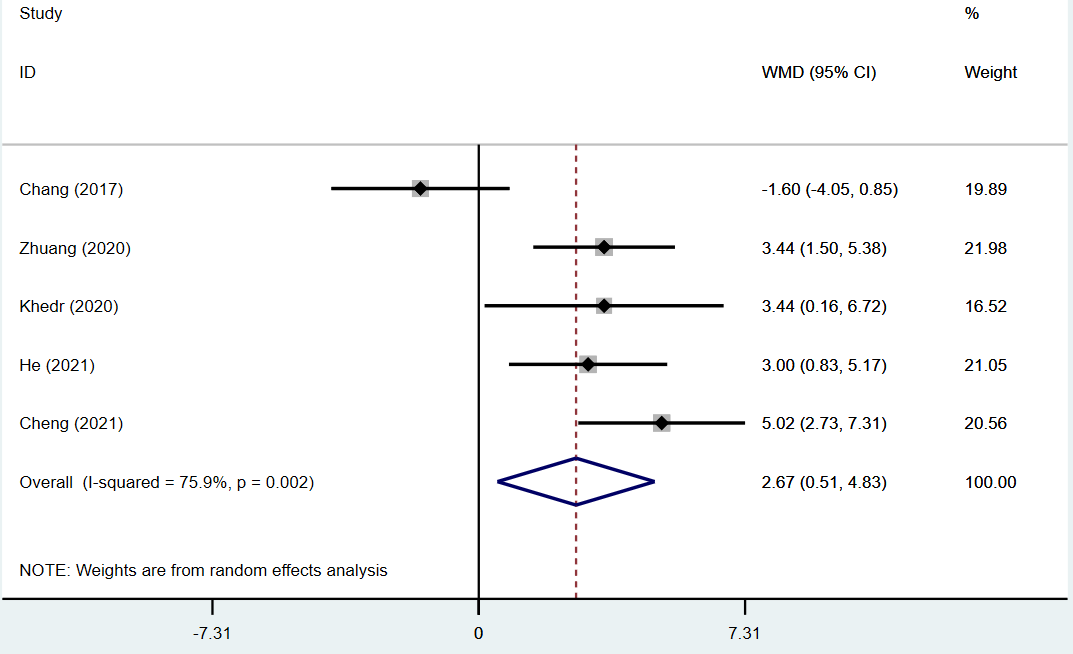
**

**Supplementary Figure S15.** Forest plot for the effect of repetitive transcranial magnetic stimulation on Montreal Cognitive Assessment

**
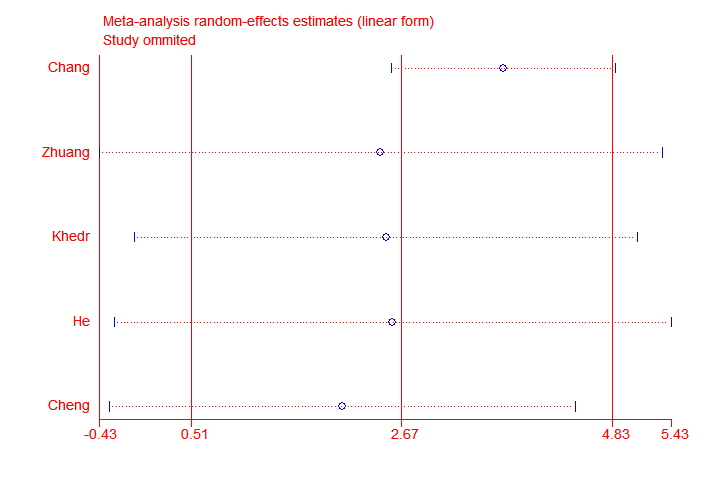
**

**Supplementary Figure S16.** The result of the sensitivity analysis of trials including Montreal Cognitive Assessment

**
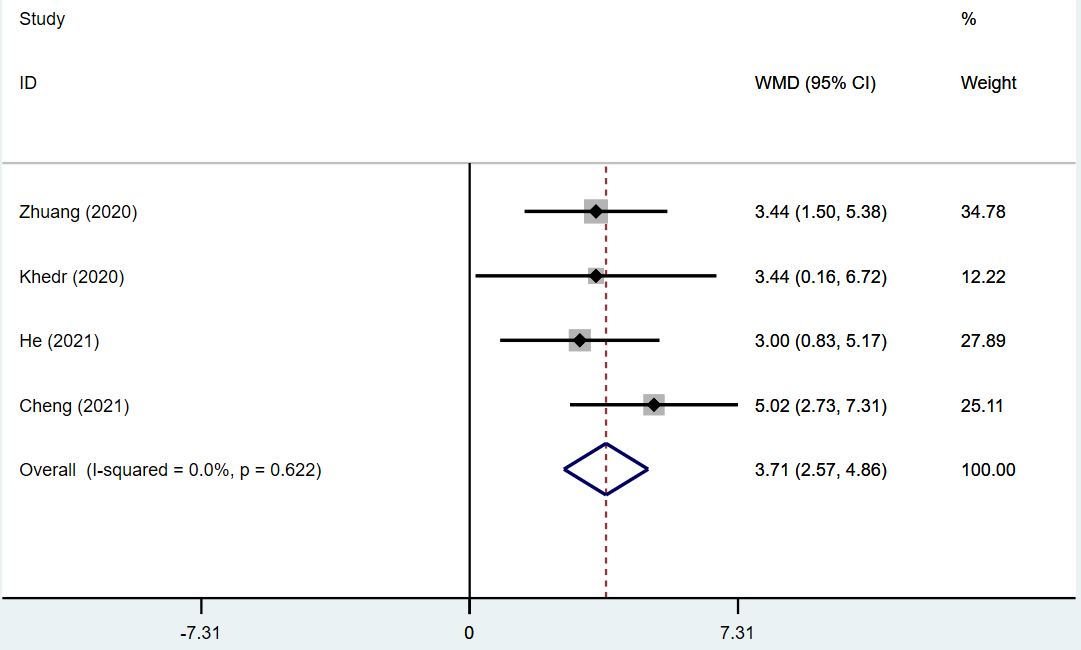
**

**Supplementary Figure S17.** Forest plot for the effect of repetitive transcranial magnetic stimulation on Montreal Cognitive Assessment after sensitivity analysis

**
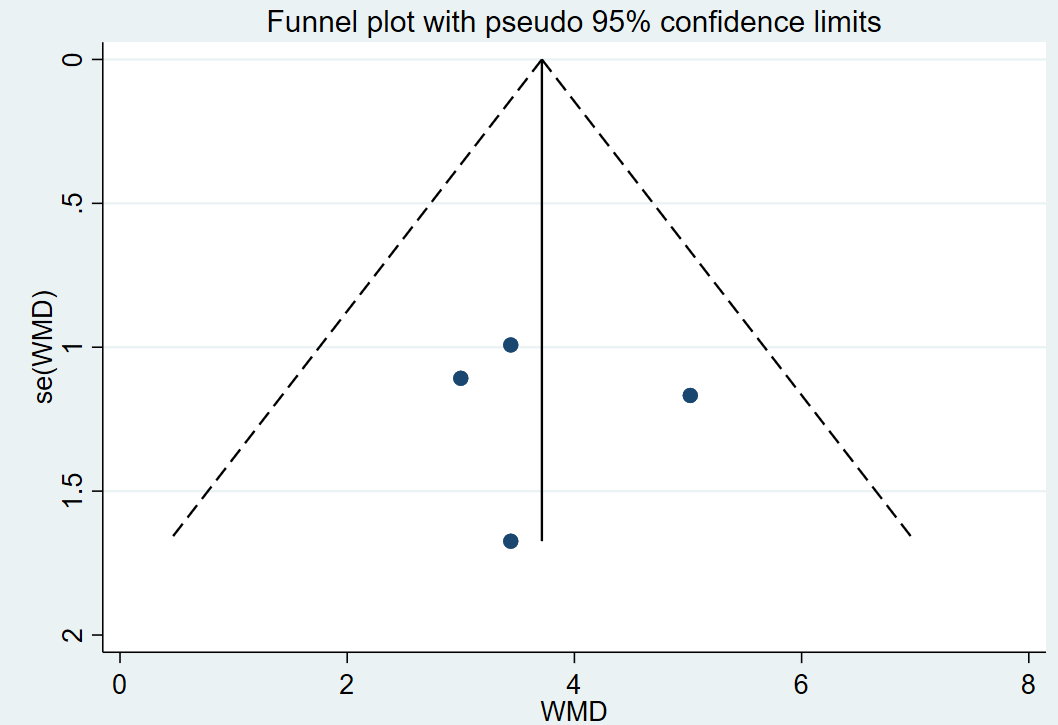
**

**Supplementary Figure S18.** Funnel plot for the effect of repetitive transcranial magnetic stimulation on Montreal Cognitive Assessment after sensitivity analysis


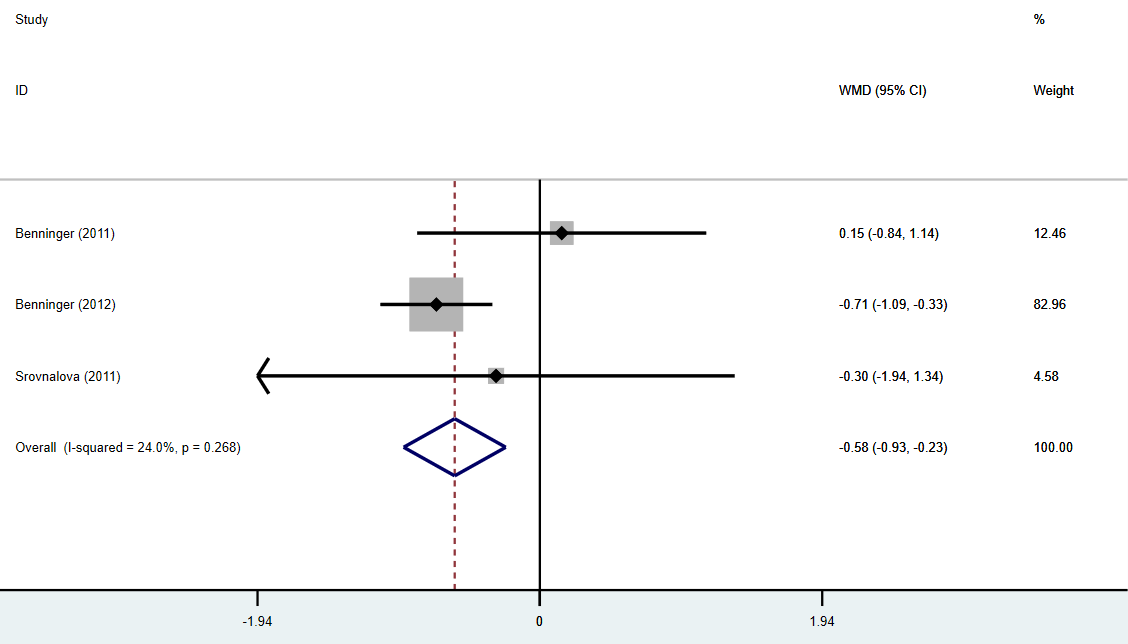


**Supplementary Figure S19.** Forest plot for the effect of repetitive transcranial magnetic stimulation on Frontal Assessment Battery


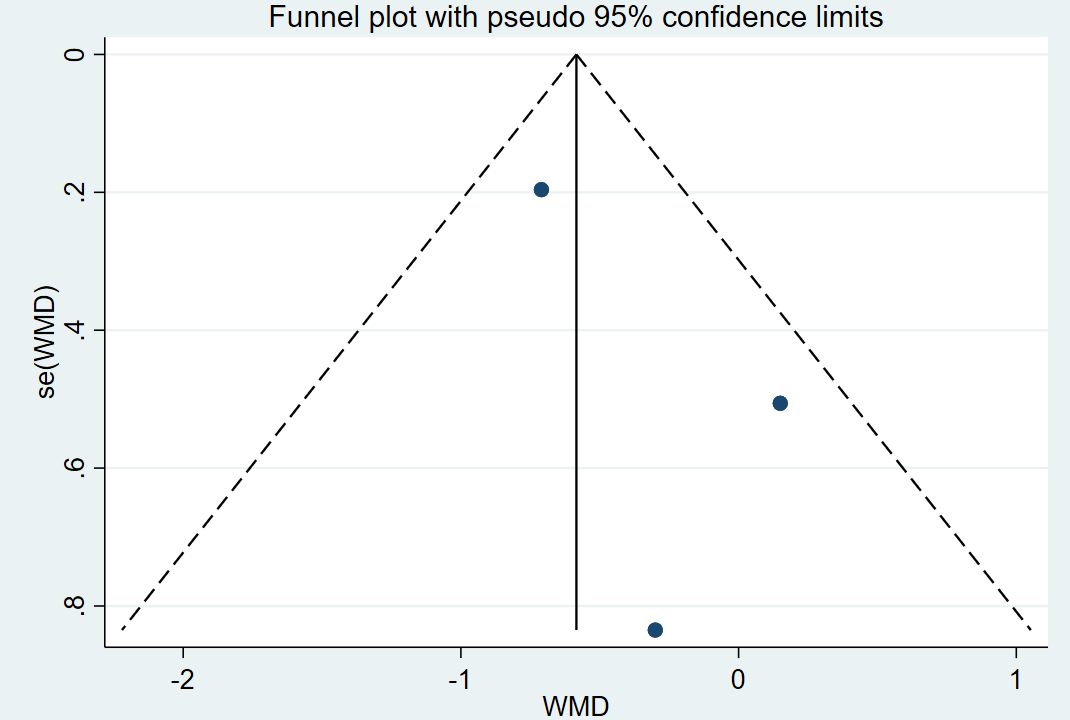


**Supplementary Figure S20.** Funnel plot for the effect of repetitive transcranial magnetic stimulation on Frontal Assessment Battery
